# Needle-warming moxibustion alleviates pain in rats with cervical spondylotic radiculopathy by modulating the NF-κB/ROS/NLRP3 pathway

**DOI:** 10.3389/fnagi.2026.1769489

**Published:** 2026-05-28

**Authors:** Bing Li, Wei Li, Xiaohui Wu, Liwei Wang, Lirong Mao

**Affiliations:** The Fourth Department of Orthopedics, The Second Affiliated Hospital of Hunan University of Chinese Medicine, Changsha, Hunan, China

**Keywords:** cervical spondylotic radiculopathy, mitochondria, needle-warming moxibustion, neuronal pyroptosis, NLRP3 inflammasome, reactive oxygen species

## Abstract

**Aim:**

This study investigates the effects of needle-warming moxibustion (NWM) on cervical spondylotic radiculopathy (CSR) by regulating the activation of the nuclear factor-kappa B (NF-κB) pathway.

**Methods:**

A CSR rat model was prepared using the fish wire extrusion method and administered postoperative treatment with NWM or injection with MitoTEMPO, with pain pressure threshold and mechanical pain threshold evaluated. Limb spasm pain-caused gait disorders were assessed using the Kawakami method. Pain mediators [Substance P (SP), Prostaglandin E2 (PGE2), and Neuropeptide Y (NPY)] and inflammatory cytokines, ROS levels, spinal cord tissue damage and positive neurons were assessed by ELISA, DHE, HE, and TUNEL staining, respectively. Serum samples from 100 CSR patients treated with NWM were collected before and after treatment. Serum inflammatory factors were determined by ELISA. Visual Analogue Scale (VAS) scores and Neck Disability Index (NDI) scores were assessed before and after treatment for CSR patients.

**Results:**

NWM increased pain thresholds and decreased SP, PGE2, and NPY protein expression and gait scores in CSR rats, indicating that NWM alleviated pain and improved motor function in CSR rats. NWM reduced inflammatory factor levels and TUNEL-positive neurons. NWM reduced ROS levels and the NLRP3 pyroptosis pathway and the NF-κB signaling axis-related protein expression in damaged tissue of CSR rats. NWM treatment reduced serum inflammatory factors and VAS and NDI scores in CSR patients. Improvements in NDI scores were positively correlated with reduced levels of IL-1β, TNF-*α*, and IL-6.

**Conclusion:**

NWM attenuates CSR by regulating the activation of the NF-κB pathway, reducing ROS, affecting the activation of the NLRP3 inflammasome, and inhibiting mitochondrial dysfunction.

## 1 Introduction

Cervical spondylosis, a prevalent condition, predominantly presents as cervical spondylotic radiculopathy (CSR), constituting approximately 60%–70% of all cases of cervical spondylosis (Luyao et al., 2022). CSR originates from degenerative alterations in the cervical intervertebral discs, hyperplasia of the posterior and articular processes, and cervical vertebral joint instability; clinically, it manifests as sensory, motor, and reflex disturbances in the cervical spinal nerve roots, particularly characterized by neck, shoulder, upper limb, and finger numbness and pain (Wang et al., 2020a). Surgical intervention for CSR aims to alleviate symptoms by decompressive measures that reduce pressure on the affected cervical nerves, yet the unpredictable distribution of cutaneous pain poses diagnostic complexities and heightens the risk of suboptimal surgical outcomes (Yang et al., 2022). Conservative management is often favored to mitigate surgical risks, and in China, both acupotomy and acupuncture have been extensively and effectively employed for CSR treatment, yielding satisfactory efficacy (Chen et al., 2019). Acupuncture, in particular, is thought to activate the body’s meridians, which are conduits for vital energy, aiming to redress imbalances and reinstate health (Patil et al., 2016). Notably, needle-warming moxibustion (NWM), a specialized acupuncture technique where a lit moxa stick is placed on the needle handle post-insertion into the acupoint, has demonstrated significant efficacy in alleviating cervical headaches and enhancing cervical mobility (Yao and Lin, 2016). Despite NWM’s promising effects, research elucidating its role and underlying mechanisms in CSR remains scarce (Yang et al., 2024a).

Furthermore, glial cells are recognized as central contributors to the development and perpetuation of neuropathic pain subsequent to nerve injury (McGinnis and Ji, 2023). The activation state of glial cells is a decisive factor in the initiation and sustenance of neuropathic pain (Zhou et al., 2023). Activated microglia, a crucial innate immune cell type within the brain, release inflammatory cytokines into the extracellular environment, causing neurotoxicity to neighboring neurons and playing a role in the etiology of numerous brain disorders (Han et al., 2021). Notably, it has been documented that the activation of the nuclear factor kappa B (NF-κB) pathway is closely linked to microglial activation (Gao et al., 2023). The NF-κB pathway plays a critical role in the regulatory mechanism of traditional Chinese medicine’s therapeutic effects on CSR (Liu et al., 2021). Additionally, improvements in CSR have been associated with elongated mitochondrial morphology in specific cell types and a reduction in the reactive oxygen species (ROS) level (Zhou et al., 2020), indicating a potential link between ROS and CSR. However, the precise mechanisms by which ROS contributes to CSR remain largely unexplored. Recent studies have identified a ROS-NLRP3 pathway involved in neuroprotection, demonstrating that the inhibition of mitochondrial ROS (mtROS)-mediated activation of the pyrin domain-containing protein 3 (NLRP3) inflammasome in microglia can prevent neuronal injury by promoting mitophagy (Han et al., 2021). Moreover, NLRP3-mediated inflammation has been implicated in a rat model of cervical spondylotic myelopathy (Liu et al., 2022). Despite these findings, research specifically addressing the NF-κB/ROS/NLRP3 axis in CSR remains scarce, highlighting the novelty of our study.

Beyond molecular mechanism exploration, our research also seeks to investigate mitochondrial dysfunction in CSR. Abnormal mitochondrial dynamics are known to play a pivotal role in the pathogenesis of various neurodegenerative diseases (Zhou et al., 2020). Notably, mitochondrial metabolic dysfunction has been observed to persist in the motor cortex of patients with cervical spondylotic myelopathy (Aleksanderek et al., 2017), further underscoring the need for a deeper understanding of mitochondrial impairment in CSR.

Synthesizing these insights, we propose the following hypothesis: NWM may ameliorate CSR by modulating the activation of the NF-κB pathway. This hypothesis is grounded in the understanding that NWM can potentially influence the neuroinflammatory environment by targeting the NF-κB pathway, thereby impacting microglial behaviors, mitochondrial function, and the subsequent neuropathic pain signaling.

## 2 Materials and methods

### 2.1 Clinical sample collection

We collected and screened 100 cases of CSR patients (61 males, 39 females, aged 48.92 ± 7.93 years, disease duration 5.2 ± 1.6 years) treated with NWM at The Second Affiliated Hospital of Hunan University of Chinese Medicine from June 2023 to December 2024. Table 1 lists detailed inclusion and exclusion criteria.

**Table 1:** Inclusion and exclusion criteria of study patients.

Inclusion criteria	1. Patients meeting the CSR diagnosis criteria outlined in the Expert Consensus on Standardized Diagnosis and Treatment of Radicular Cervical Spondylosis, presenting with neck and shoulder pain accompanied by radiating pain or numbness in the upper limbs, positive Spurling’s test, and imaging studies (X-ray/MRI) demonstrating intervertebral foramen stenosis or nerve root compression
	2. Patients diagnosed with Qi Stagnation and Blood Stasis syndrome based on the Clinical Practice Guidelines in Traditional Chinese Medicine Rehabilitation-Cervical Spondylosis (Xiangbi), manifesting symptoms including stabbing pain in the neck and shoulders, fixed tender points that are averse to pressure, limb numbness, pale red or dark purple tongue with ecchymoses, and taut and hard pulse
	3. Patients aged 18–65 years with a visual analogue scale (VAS) score ≥ 4 who have not received other systemic treatments within the past month
Exclusion criteria	1. Other types of cervical spondylosis (e.g., myelopathic, sympathetic, or mixed types)
	2. Concurrent cervical spine fractures, tumors, tuberculosis, or severe osteoporosis
	3. Pregnancy or lactation
	4. History of cervical spine surgery or epidural injections within the past month
	5. Concurrent psychiatric disorders or severe systemic diseases involving vital organs such as the heart, brain, or lungs.
	6. presence of skin lesions or infections at the acupuncture site

Blood samples (2 mL) were collected from the patient’s elbow vein in a fasting state before treatment and 15 days after treatment. After standing at room temperature for 30 min, the samples were centrifuged to separate the supernatant serum, which was stored at −80 °C. At the same time points, pain intensity was assessed using the visual analogue scale (VAS), and cervical spine function was evaluated using the Neck Disability Index (NDI). Adverse reactions were closely monitored during treatment. Two patients exhibited mild, transient local erythema at the acupoints; no other serious complications such as burns were recorded. Written informed consent was obtained from all participants, and the study was conducted in accordance with the *Declaration of Helsinki* and approved by The Second Affiliated Hospital of Hunan University of Chinese Medicine Ethics Committee, with the ethical review license number 2024-KY-16.

### 2.2 Treatment protocol

For needles and moxibustion tools, we used 0.35 mm × 40 mm stainless steel filiform needles from Huatuo brand (ShunTeng Biotechnology, Jinan, China) and selected 5-year-old mugwort (Nanyang LaoAiLing, China). The main acupoints included Fengchi (GB20), C3-6 Jiaji, and Dazhui (GV14) and the auxiliary acupoints included Quchi (LI11) and Hegu (LI4) when upper limb pain was present.

When needling Fengchi, the direction was obliquely towards the nose tip with a depth of 0.8 to 1.2 inches; for C3-6 Jiaji, the direction was direct with a depth of 0.5 to 1.0 inches; for Dazhui, the direction was obliquely towards the affected side at 45° with a depth of 0.5 to 1.2 inches. The needling sensation should radiate to the shoulder and back on the affected side. For the upper limbs’ Quchi and Hegu acupoints, the needle was inserted directly toward the affected side at a depth of 0.5 to 1.0 inches. After obtaining the needling sensation, moxibustion was performed by igniting the mugwort on the needle handle from below, with a hard piece of paper placed in the acupoint area, and 3 to 5 moxa cones per session to avoid burning the patients’ skin. The treatment duration, frequency, and course were approximately 30 min per session, once a day, for three consecutive weeks.

### 2.3 Scoring observations

VAS scores were measured using a 10-cm sliding ruler, with the “0” end indicating no pain and the “10” end indicating severe pain. Patients were asked to mark their pain levels before and after treatment, with a higher number being indicative of more severe pain.

NDI scores assessed the intensity of shoulder and neck pain in patients with CSR, and the impact of pain on personal life care, daily sleep, entertainment, and work ability. The scale consists of 10 questions, each with 6 outcome options, scored from 0 to 5 points, with a higher score indicating more severe cervical spine dysfunction.

### 2.4 Experimental animals

Specific pathogen free-grade Sprague–Dawley male rats (weight: 250 ± 20 g) were purchased from Beijing Vital River Experimental Animal Technology Co., Ltd., with an animal license number of [SYXK (Beijing) 2022–0052]. Rats were housed in a sterile environment at 26–28 °C with 40%–60% humidity, provided with free access to food and water, and maintained in a 12-h light/dark cycle. All animal experiments in this study were reviewed and approved by The Second Affiliated Hospital of Hunan University of Chinese Medicine’s Animal Ethics Committee. (Approval number: LLBH-202311070022).

### 2.5 Establishment of the CSR rat model

A CSR rat model was established using Sprague–Dawley rats by applying filament compression to the left cervical nerve root (Su et al., 2023). Rats were fasted and deprived of water for 12 h prior to the procedure and were anesthetized with an intraperitoneal injection of 2% sodium pentobarbital solution at 0.2 mL/100 g. The rats were secured in a prone position, and the neck area was prepared with a razor. After routine disinfection, the highest thoracic vertebrae (T2) were located by touch. An approximately 3 cm incision was made upwards along the midline of the neck. Subcutaneous tissue and posterior neck muscles were separated to expose the left lateral arch from C6 to T2, with surface muscles and ligaments scraped off. Subsequently, the connective tissue and ligamentum flavum at the C6/C7 and C7/T1 intervertebral spaces were cut with micro-forceps. The left arch of C7 was gently opened with pointed mosquito forceps. A nylon filament (approximately 1.5 cm long and 0.5 mm in diameter) was placed under the nerve roots from C6 to T1 along the longitudinal axis of the spinal cord using microsurgical forceps to compress the nerve roots. The sham group underwent the same surgical exposure without spinal canal insertion.

A total of 84 SD rats were included in this study and randomly divided into 7 groups, with 12 rats in each group (Table 2). Each group of 12 rats included 6 animals for morphological analysis [hematoxylin and eosin (HE) staining, terminal deoxynucleotidyl transferase dUTP nick-end labeling (TUNEL) staining, immunohistochemistry (IHC), immunofluorescence, etc.] and 6 animals for molecular testing (Western blot, ELISA, ROS detection, mitochondrial function assessment, etc.), with no overlap between the two sets of data. The final sample size included in statistical analysis was 6 rats per group (6 for morphological and 6 for molecular experiments), with all data derived from independent samples (Kullmann et al., 2019).

**Table 2:** Grouping and treatment of animals.

Groups	Treatments	Intervention (beginning 7 days post-surgery)
Sham group	Exposure only; no spinal canal insertion	No
CSR group	Filament compression of the C6-T1 nerve root	No
NWM group	Filament compression of the C6-T1 nerve root	Warm acupuncture intervention, once daily, for 7 consecutive days
Sham + NS group	Exposure only; no spinal canal insertion	An equal volume of saline solution was administered intraperitoneally once daily for 1 week.
Sham + MT group	Exposure only; no spinal canal insertion	0.7 mg/kg of MitoTEMPO was administered intraperitoneally once daily for 1 week
CSR + NS group	Filament compression of the C6-T1 nerve root	An equal volume of saline solution was administered intraperitoneally once daily for 1 week.
CSR + MT group	Filament compression of the C6-T1 nerve root	0.7 mg/kg of MitoTEMPO was administered intraperitoneally once daily for 1 week

NWM treatment: After securing rats in the restraint apparatus, acupoint localization and intervention were performed. Based on the Atlas of Acupoints for Animals and the Experimental Acupuncture Manual, the following points were selected: Fengchi (GB20), Jiaji (C3-C6), and Dazhui (GV14). Hair was scraped clean to expose the acupoints. Filiform needles (0.25 mm × 25 mm) were used for needling. Five-year-old mugworts (Nanyang LuYing, China) were used. After iodine-based disinfection, needles were inserted. Moxa cones (1.2 cm × 1.5 cm) were applied atop the needle handles at all three points. Cones were replaced promptly upon burnout, completing three moxa applications per session. Each session lasted 15 min, administered once daily for seven consecutive days.

After the 2-week experiment, rats were anesthetized with 2% pentobarbital sodium (80 mg/kg). They were processed as follows: For morphological detection (6 rats per group), the left ventricle was rapidly perfused with 150–200 mL of pre-chilled normal saline to flush out intravascular blood. Once the effluent became clear and the liver appeared pale, approximately 200 mL of 4% paraformaldehyde solution was perfused for thorough fixation. Once rigor mortis set in, the enlarged cervical spinal cord segment (C5-T1) was immediately harvested and placed in 4% paraformaldehyde for fixation and storage. For rats used in molecular assays (6 per group), blood samples were first collected via the abdominal aorta (serum separated by routine centrifugation and stored at −80 °C). Immediately thereafter, spinal cord tissue from the cervical enlargement segment was rapidly harvested and stored in liquid nitrogen or at −80 °C for subsequent use.

### 2.6 Pressure pain threshold (PPT)

The YLS-3E type pain analyzer (ZS Dichuang, Beijing, China) was used to measure the PPT of rats before and after intervention. A blunt tip (resin glass cone) was applied between the third and fourth metatarsal bones of the left front foot, gradually increasing linear pressure until the rat screamed and struggled to pull its foot back. To prevent tissue damage, the cutoff pressure was set at 1000 g based on preliminary experimental results. If the pressure reached this value and the rat still showed no response, stimulation was terminated and 1,000 g was recorded as the threshold for that measurement. The average value was taken from three consecutive measurements.

### 2.7 Mechanical pain threshold (MPT)

Rats were placed in a Plexiglass cage with a metal mesh at the bottom and allowed to acclimate for 15 min. A set of von Frey filaments (North Coast, United States) was then used to measure the MPT of the plantar area of the left forelimb, and the rat’s paw withdrawal or licking response was observed. If there was no response, it was marked as “O”; otherwise, it was marked as “X.” If an “OX” or “XO” combination occurred, four additional measurements were required to obtain a sequence of “O” and “X.” The MPT was calculated using the following formula mentioned in previous literature: MPT_(g)_ = (10^(Xf + kδ)^)/10,000 (Xf: the number of the last filament; u: the average difference between the filaments, approximately 0.224; k: the table reference value according to the above sequence) (Deuis et al., 2017; Yang et al., 2023).

### 2.8 Behavioral tests

Rats were placed in an observation box and allowed to acclimate for 30 min before observation of their behaviors, such as licking or squeaking. Gait disorders caused by limb spasm pain were assessed using the Kawakami method (Cai et al., 2020) which was discussed before (1 point: left forelimb without flexion deformity, normal gait; 2 points: left forelimb with inward flexion foot deformity, slight or unable to hold weight, walking limp; 3 points: in the presence of inward flexion bending foot deformity in the left forelimb, walking without touching the table). A gait score of ≥ 2 points was seen as the criterion for successful modeling (Cai et al., 2022).

### 2.9 Enzyme-linked immunosorbent assay (ELISA)

According to the manufacturer’s protocol, IL-1 beta (ab255730), IL-6 (ab234570), TNF-*α* (ab236712), and IL-18 (ab213909) levels were determined using respective ELISA kits from Abcam (Cambridge, UK). Additionally, Substance P (SP; E-EL-0067), Prostaglandin E2 (PGE2; E-EL-0034), and Neuropeptide Y (NPY; E-EL-R0655) levels were measured using ELISA kits from Elabscience. The experiment was independently repeated three times.

### 2.10 Histopathological examination

Spinal cord tissue was examined histologically using HE staining method. Tissue samples after perfusion were placed in a 4% paraformaldehyde solution, dehydrated in an ethanol gradient, embedded in paraffin, and cut into 4 μm thick sections. After deparaffinization, sections were stained with an HE staining kit (G1120, Solarbio, Beijing, China) and sealed with neutral resin after gradient alcohol dehydration. Samples were observed and photographed under an Olympus BX53 optical microscope (Olympus, Tokyo, Japan), and the area of tissue damage and inflammatory cell density in the damaged area were quantitatively analyzed using Image J software (Media Cybernetics, United States).

### 2.11 Immunofluorescence

Tissue sections were rinsed with PBS and blocked with PBS containing 5% bovine serum albumin and 0.3% Triton X-100. Subsequently, sections were incubated overnight at 4 °C with the following primary antibodies: neuronal nuclei antigen (NeuN; #24307, 1:100, CST, Danvers, MA, USA), phosphorylated NF-κB p65 (#3033, 1:200, CST), or Iba1 (#58410, 1:200, CST). After three washes with PBS, samples were imaged using secondary antibodies Alexa Fluor® 488-conjugated Anti-mouse IgG (H + L; #94207, CST), Alexa Fluor® 594-conjugated Anti-rabbit IgG (H + L; #8889, CST), or TUNEL detection solution (C1088; Beyotime, Shanghai, China), and observed under a DMI3000 B fluorescence microscope (Leica, Wetzlar, Germany). Using ImageJ software, 3–5 random fields of view were selected in the dorsal horn region of each spinal cord section. The average fluorescence intensity of Iba-1 or p-NF-κB p65 was measured to reflect changes in their protein expression levels.

### 2.12 Western blotting (WB)

Total protein from spinal cord tissue was extracted on ice using radioimmunoprecipitation assay lysis buffer (P0013E, Beyotime). The supernatant was collected, and protein concentration was determined using a bicinchoninic acid protein assay kit (P0011, Beyotime). Proteins were separated by 10% Sodium Dodecyl Sulfate-Polyacrylamide Gel Electrophoresis and transferred onto Polyvinylidene Fluoride membranes. Membranes were blocked in Tris-buffered saline with Tween-20 (TBST; 20 mM Tris, 137 mM NaCl, 0.1% Tween-20) containing 5% skim milk for 1 h. Primary antibodies *α*-syn (#4179, 1:1000, CST), synapsin1 (#5297, 1:1000, CST), synapsin2 (#85852, 1:1000, CST), NLRP3 (#15101, 1:1000, CST), caspase1 (#24232, 1:1000, CST), ASC (#67824, 1:1000, CST), GSDMD (#39754, 1:1000, CST), IKBa (4,812, 1:1000, CST), p-IKBa (#2859, 1:1000, CST), phosphate-p65 (#3033, 1:1000, CST), and p65 (#8242, 1:1000, CST) were added for incubation overnight at 4 °C. After washing, membranes were incubated with secondary antibody Goat Anti-Rabbit IgG antibody (HRP; #35401, 1:2000, CST) for 2 h at room temperature, with glyceraldehyde 3-phosphate dehydrogenase (GAPDH; #2118, 1:1000, CST) as the loading control. Protein bands were detected using an ECL Plus chemiluminescence kit (Life Technology, Beijing, China), and densitometry analysis was performed using Image J software (Media Cybernetics, United States).

### 2.13 IHC

After paraffin embedding, tissue sections were subjected to high-temperature and high-pressure antigen retrieval for 2 min and blocked in goat serum (SL038, Solarbio) for 20 min at room temperature. Sections were incubated overnight at 4 °C with anti-PSD-95 (#3409, 1:100, CST) and anti-GAP-43 (ab75810, 1:50, Abcam) antibodies, followed by incubation with goat anti-rabbit antibody IgG H&L (HRP; ab214050, 1:1000, Abcam) for 60 min at 37 °C. Sections were then stained with 3,3’-Diaminobenzidine (DA1016, Solarbio) and counterstained with hematoxylin (H8070, Solarbio), and images were captured under the DMI3000 B fluorescence microscope (Leica). Using ImageJ software, three random fields of view were selected from each section, with the percentage of positive staining area relative to the total field of view serving as the quantitative metric.

### 2.14 Dihydroethidium (DHE) staining

To assess ROS levels in spinal cord tissue, cervical spinal cord sections were stained with DHE. DHE powder was dissolved in dimethyl sulfoxide (DMSO). Frozen sections were incubated with the DHE solution at room temperature for 30 min, and fluorescence was analyzed using the DMI3000 B fluorescence microscope (Leica).

### 2.15 MitoSOX fluorescence for mitochondrial ROS (mtROS)

MitoSOX Red (S0061S, Beyotime) was used to determine the production of mitochondrial-derived ROS as described previously (Ye et al., 2016). Briefly, MitoSOX Red was dissolved in a mixture of DMSO and saline at 1:1, with a final concentration of 33 μM. Under sodium pentobarbital anesthesia, rats were injected intrathecally with 20 μL of MitoSOX Red. After perfusion, the spinal cord was removed, fixed in 4% paraformaldehyde for 2 h, and then placed in 30% sucrose at 4 °C for 48 h. Spinal cord sections (25 μm) were cut in a cold chamber and observed under the DMI3000 B fluorescence microscope (Leica). Image J software (Media Cybernetics, United States) was used to analyze the intensity of MitoSOX-positive cells in the spinal cord dorsal horn.

### 2.16 Annexin V-PE/7-AAD staining

Tissue was prepared into a single-cell suspension, washed with PBS, centrifuged, and resuspended in buffer. Then, 5 μL of Annexin V-PE and 5 μL of 7-AAD were added to 400 μL of cell suspension and incubated at room temperature in the dark for 15 min. Samples were analyzed using a flow cytometer (Aceabio, San Diego, CA, United States), and the percentages of various cell subpopulations were analyzed using FlowJo 7.6 software.

### 2.17 Detection of cell membrane potential by JC-1

Changes in mitochondrial membrane potential in spinal cord tissue were determined using the JC-1 mitochondrial membrane potential assay kit (C2006, Beyotime). Tissue was first prepared into a single-cell suspension, and the JC-1 fluorescence probe was assembled according to the kit instructions, and images obtained from a fluorescence microscope. At high mitochondrial membrane potential, JC-1 aggregates in the mitochondrial matrix to form polymers, emitting red fluorescence. At low mitochondrial membrane potential, JC-1 does not aggregate in the mitochondrial matrix, and green fluorescence is observed. Changes in mitochondrial membrane potential were detected based on changes in the fluorescence color.

### 2.18 Ca^2+^ concentration detection

Fluo-3 a.m. (S1056, Beyotime) was used to detect intracellular free Ca^2+^ levels. Briefly, tissue was prepared into a single-cell suspension and incubated with Fluo-3 a.m. at 37 °C for 1 h. The cells were then washed three times with PBS, and absorption values of all wells were measured using a microplate reader (Tecan Infinite F50, Switzerland) at 488 nm excitation and 530 nm emission. Fluorescence values were substituted into a standard curve to obtain Ca^2+^ concentrations. At least three independent replicate experiments were set up for each group.

### 2.19 RT-qPCR for mitochondrial DNA levels (mtDNA)

This study used a DNA extraction kit (DP304, Tiangen Biotechnology, Beijing, China) to extract total DNA from tissue samples. Primer sequences for ND1 and GAPDH were synthesized by Sangon Biotech Co.

The primer sequences are: ND1 (Forward: GTCACAATAGCCATTATCCTC, Reverse: CTGATTCTCCTTCTGTTAAGTC); GAPDH (Forward: GGAAGGACTCATGACCACAGT, Reverse: GCCATCACGCCACAGTTTC), and qPCR was used to detect mtDNA and nuclear DNA (nDNA). The ratio of mtDNA to nDNA was used to represent the relative mtDNA content, with ND1 and GAPDH representing mtDNA and nDNA, respectively.

### 2.20 Statistical analysis

All data were analyzed and graphed using GraphPad Prism 9.5.0 (GraphPad Software Inc., San Diego, CA, United States). The Shapiro–Wilk test was used to assess normal distribution. Data were presented as the mean ± standard deviation. Comparisons between two groups were made using the independent sample *t*-test, while multiple group comparisons were conducted using one-way analysis of variance (ANOVA), followed by Tukey’s multiple comparison test for post-hoc analysis. A *p*-value of less than 0.05 was considered to indicate statistically significant differences.

## 3 Results

### 3.1 NWM alleviates pain behaviors in CSR rats

**Figure 1:**
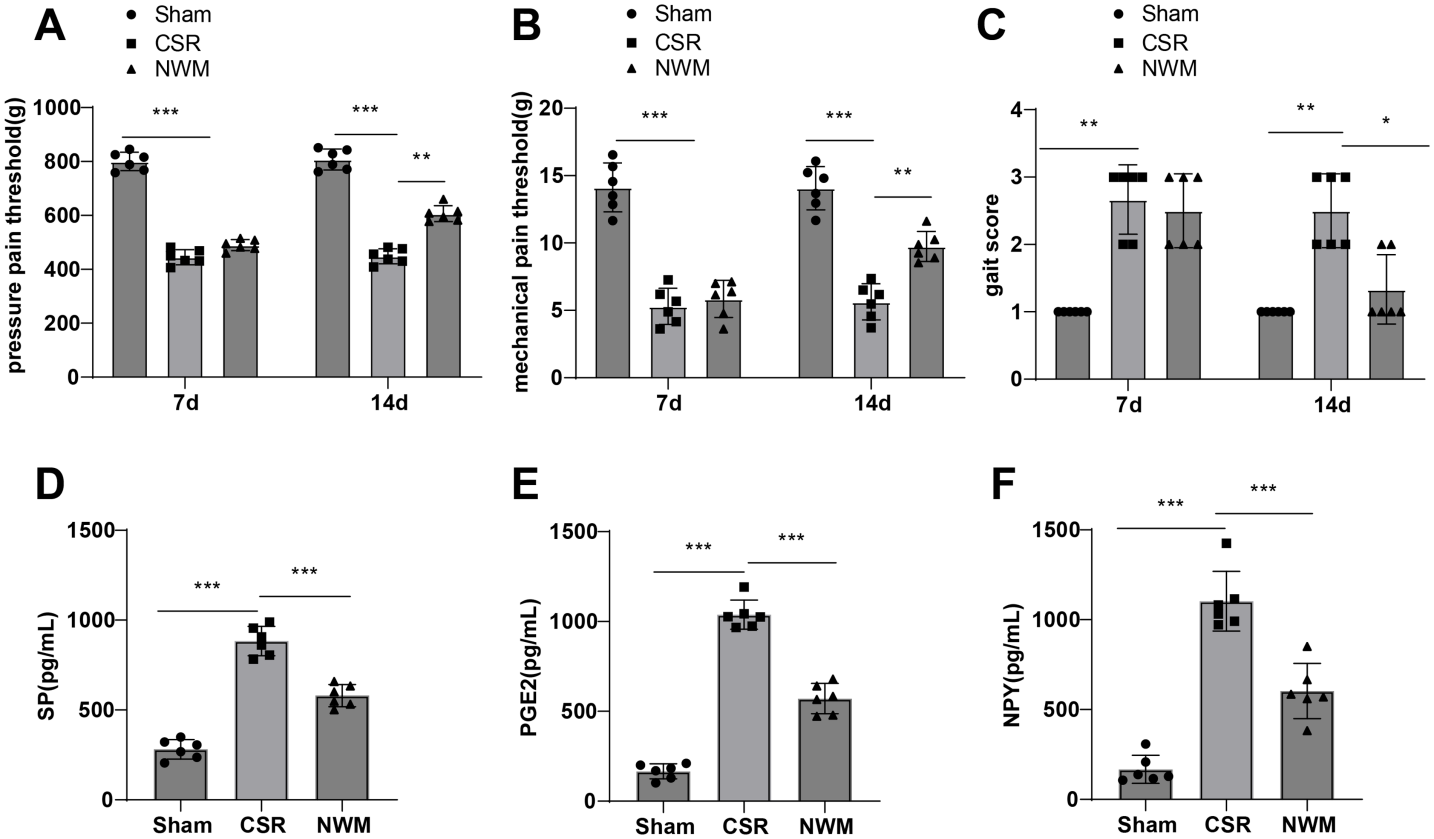
Improvement of pain behavior and downregulation of pain mediator expression in CSR rats by NWM. **(A)** PPT measured using YLS-3E electronic pressure meters; **(B)** MPT determined by Von Frey filaments; **(C)** Gait scores of rats before and after intervention; **(D–F)** ELISA detections of SP, PGE2, and NPY contents in cervical nerve roots. *n* = 6. Data were presented as mean ± standard deviation, with multiple group comparisons performed using one-way ANOVA, followed by Tukey’s multiple comparison test. * *p* < 0.05, ** *p* < 0.01, *** *p* < 0.001.

To investigate the analgesic effects of NWM on CSR rats, the YLS-3E electronic pressure meter and Von Frey filaments were used to measure the rats’ PPT and MPT. Results revealed that PPT and MPT in CSR model rats were significantly lower than those in the Sham group (*p* < 0.001). Following NWM intervention, PPT and MPT in CSR rats markedly recovered (*p* < 0.001) (Figures 1A,B). Consistent with this behavioral finding, gait scores assessing forelimb motor function were elevated in the CSR group compared to the Sham group (*p* < 0.001) (Figure 1C), indicating impaired motor function. NWM treatment effectively improved this impairment, with gait scores significantly lower than those in the CSR group (*p* < 0.05) (Figure 1C). To investigate the molecular basis of these behavioral improvements, key pain mediators SP, PGE2, and NPY in spinal cord tissues were measured. ELISA analysis revealed that SP, PGE2, and NPY levels in the CSR group were significantly higher than those in the Sham group (*p* < 0.001). NMW treatment reduced CSR-induced SP, PGE2, and NPY protein expression (*p* < 0.05) (Figures 1D–F). Collectively, these findings indicate that CSR treatment increases the release of pain mediators, while NMW can mitigate this phenomenon and alleviate pain-related behaviors induced by CSR.

### 3.2 NWM alleviates inflammatory responses and neuronal damage in CSR rats

**Figure 2:**
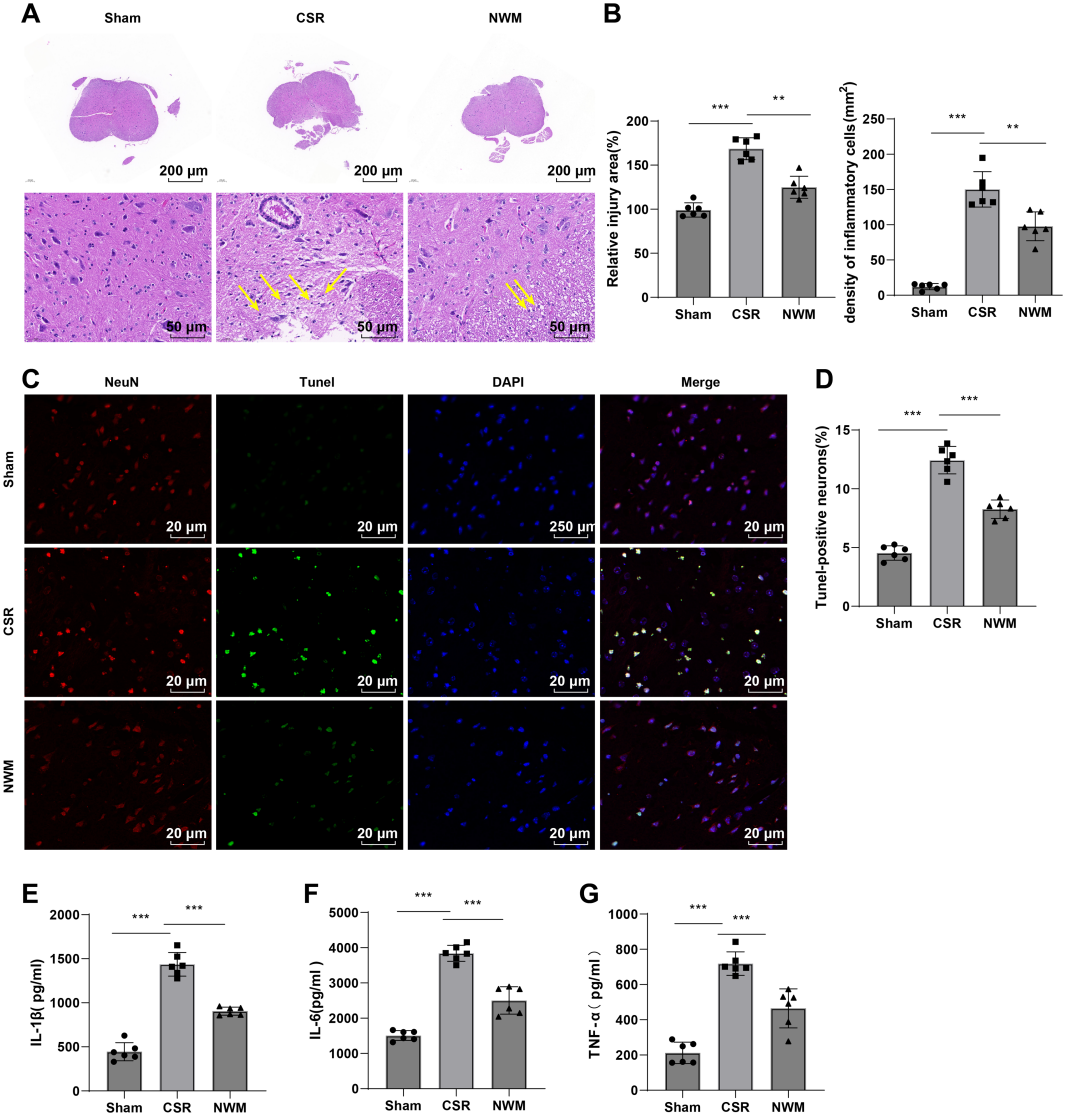
Alleviation of inflammatory responses and neuronal damage in CSR rats caused by NWM. **(A,B)** Quantitative analysis of tissue damage and inflammatory cell density in injured areas observed by HE staining (Arrows indicate inflammatory cells); **(C,D)** TUNEL staining and quantification of positive neurons; **(E–G)** ELISA detection of inflammatory factors IL-1β, IL-6, and TNF-*α* levels. *n* = 6. Data were presented as mean ± standard deviation, with multiple group comparisons performed using one-way ANOVA and post-hoc Tukey’s multiple comparison test. * *p* < 0.05, ** *p* < 0.01, *** *p* < 0.001.

The aforementioned behavioral and pain mediator findings indicate that NWM effectively alleviates pain symptoms in CSR. Given that inflammatory response is a key factor in CSR pathogenesis (Karadimas et al., 2013), further analysis was conducted on inflammatory reactions, neuronal damage, and synaptic plasticity changes in spinal cord tissue. It is hypothesized that the improvement in pain behavior may be closely related to the anti-inflammatory and neuroprotective effects of NWM on the local microenvironment of the nerve root. Histopathological examination of spinal cord tissue using HE staining revealed increased tissue damage and inflammatory cells in the injured region of CSR rats. NWM reduced tissue damage and inflammatory cells in the injured region of CSR rats (*p* < 0.001) (Figures 2A,B). Neuronal damage, as a key determinant of neurological deficits, was assessed through TUNEL staining to evaluate neuronal apoptosis (Karadimas et al., 2013). Results showed an increase in TUNEL-positive neurons in CSR rats (*p* < 0.001), while NWM treatment significantly reduced neuronal apoptosis in CSR rats (*p* < 0.001) (Figures 2C,D). ELISA detection revealed that CSR rats exhibited elevated levels of inflammatory cytokines IL-1β, IL-6, and TNF-*α* compared to Sham rats (all *p* < 0.001), whereas NWM treatment reduced cytokine release levels in CSR rats (all *p* < 0.001) (Figures 2E–G).

**Figure 3:**
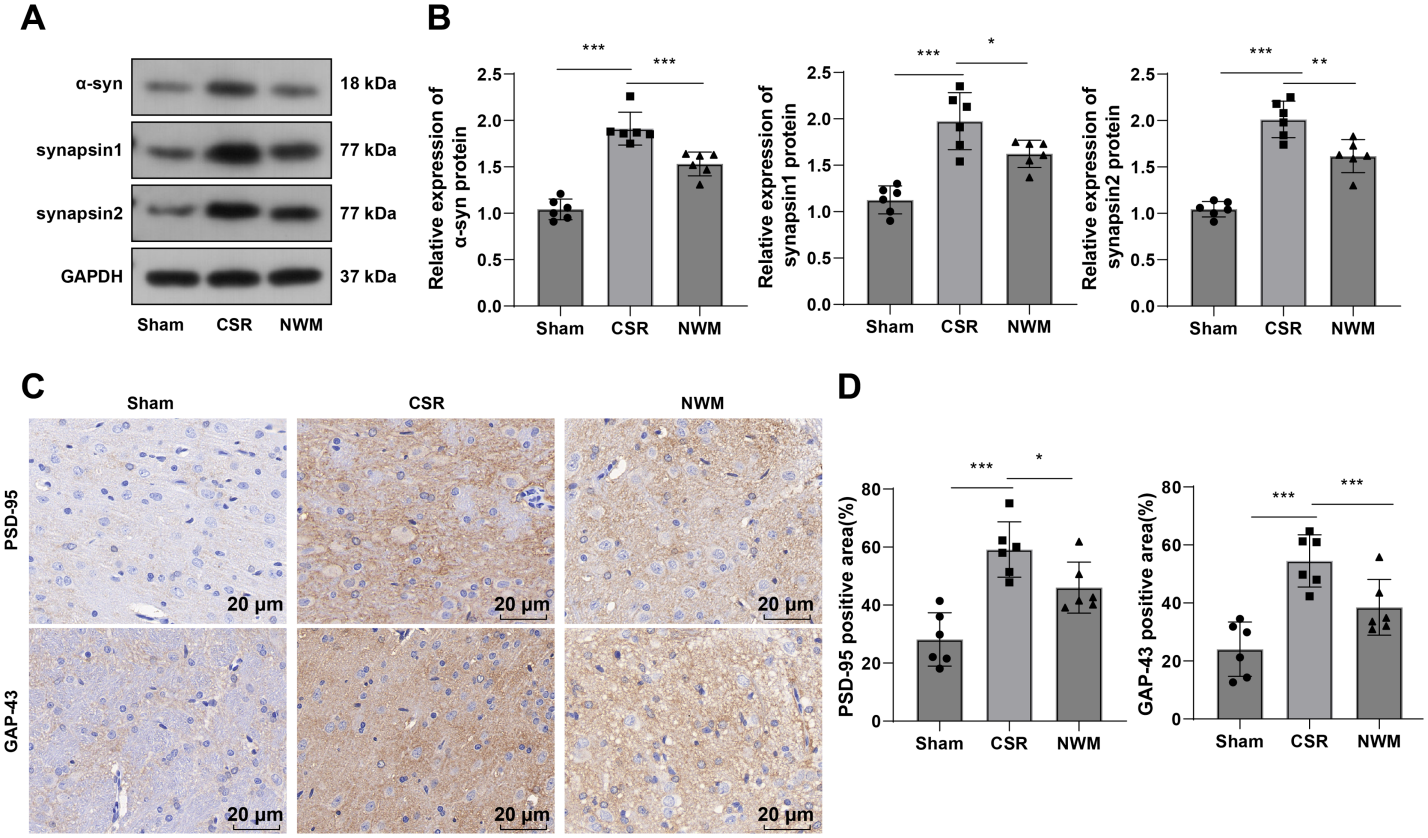
Improvement of synaptic plasticity in CSR rats by NWM. **(A,B)** Representative immunoblot images and quantitative analysis of α-syn, synapsin1, and synapsin2 protein expression detected by WB; **(C,D)** Expression of PSD-95 and GAP-43 detected by IHC. *n* = 6. Data were presented as mean ± standard deviation, with multiple group comparisons performed using one-way ANOVA and post-hoc Tukey’s multiple comparison test. *p < 0.05, ** *p* < 0.01, *** *p* < 0.001.

Given that synaptic proteins such as α-syn, synapsin1, synapsin2, PSD-95, and GAP-43 are important markers for detecting synaptic plasticity and play a crucial role in the occurrence of pain hypersensitivity (Yang et al., 2023), the effects of NWM on the expression of these synaptic proteins were further investigated. WB detection showed that α-syn, synapsin1, and synapsin2 protein expression in the cervical spinal cord of rats in the CSR group was higher than that in the Sham group (all *p* < 0.001), while the NWM group exhibited lowered expression compared to the CSR group (all *p* < 0.05) (Figures 3A,B). IHC analysis of postsynaptic markers PSD-95 and GAP-43 revealed that spinal cord levels of both proteins were significantly elevated in the CSR group compared to the Sham group (all *p* < 0.001), while the levels of PSD-95 and GAP-43 in the spinal cord of NWM-treated rats were lower than those in the CSR group (all *p* < 0.05) (Figures 3C,D). These results indicate that NWM exerts a positive effect on inflammatory responses and neuronal damage in CSM rats, while also enhancing synaptic plasticity. This may represent one of its key mechanisms for alleviating pain.

### 3.3 NWM inhibits the activation of the NF-κB pathway and NLRP3 inflammasome in CSR rats

**Figure 4:**
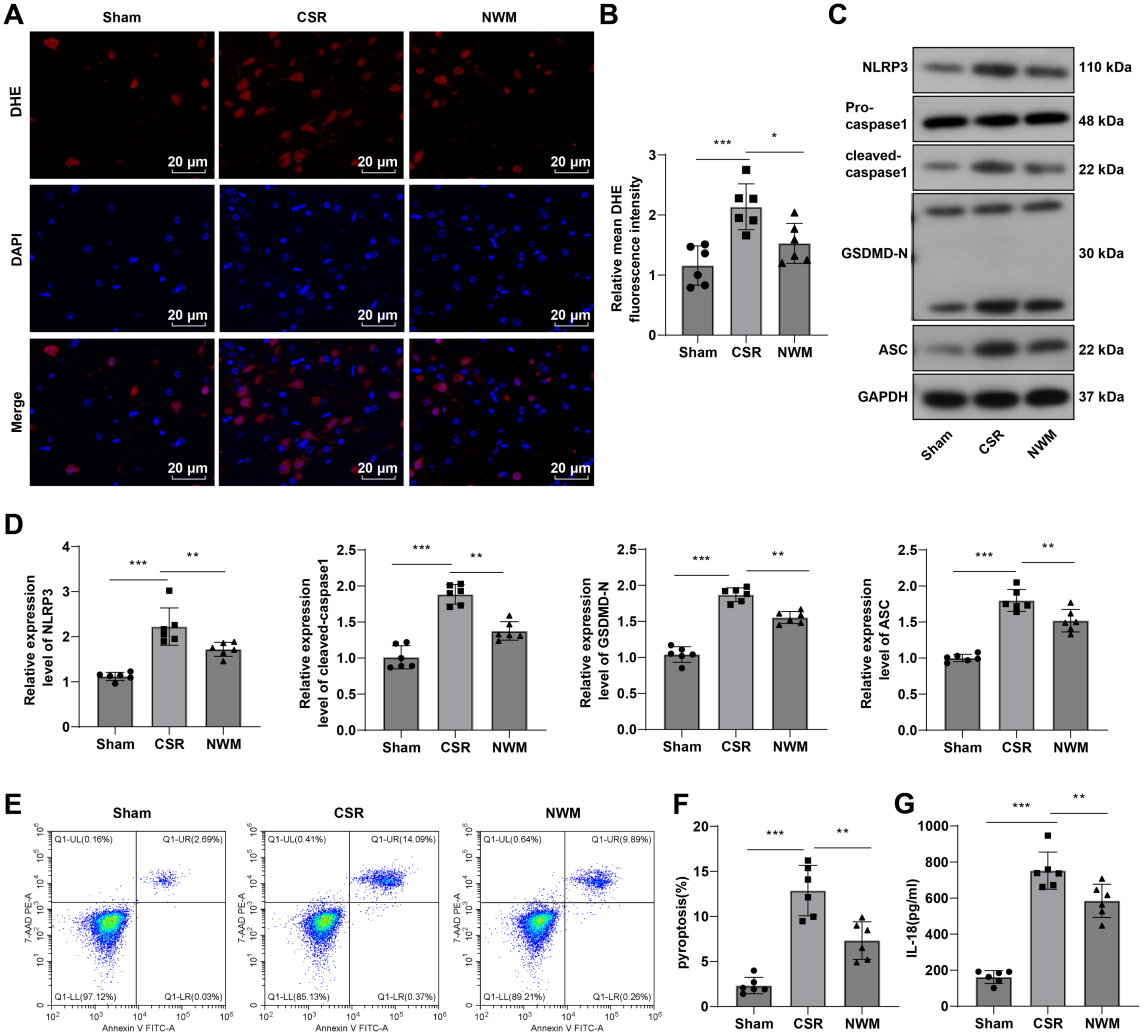
Inhibition of NLRP3 inflammasome activation in CSR rats by NWM. **(A,B)** DHE staining assay for measuring ROS levels in cervical spinal cord tissue; **(C,D)** Representative immunoblot images and quantitative analysis of NLRP3, pro-caspase-1, cleaved-caspase1, ASC, and GSDMD-N protein expression determined by WB; **(E,F)** Detection of cervical spinal cord tissue pyroptosis by flow cytometry (Annexin V-PE and 7-AAD double staining method); **(G)** ELISA detection of the inflammatory factor IL-18. *n* = 6. Data were presented as mean ± standard deviation, with multiple group comparisons performed using one-way ANOVA and post-hoc Tukey’s multiple comparison test. * *p* < 0.05, **p < 0.01, ***p < 0.001.

Dysregulation of inflammatory responses is one of the important factors causing damage, often accompanied by changes in stress responses within the body. Therefore, the level of ROS in the cervical spinal cord tissue was measured using the DCFH-DA staining assay, showing increased ROS levels in the injured area of CSR rats compared to the Sham group (*p* < 0.001). NWM treatment reduced ROS levels in the injured area of CSR rats (*p* < 0.001) (Figures 4A,B). Concurrently, high levels of ROS can lead to the activation of NLRP3 inflammasomes, a key factor triggering inflammatory responses (Gu et al., 2019; Ma et al., 2021). Therefore, NLRP3-mediated pyroptosis was further investigated. GSDMD-N is the *N*-terminal active fragment of the Gasdermin D protein. Upon activation of the NLRP3 inflammasome, caspase-1 cleaves the GSDMD protein, releasing its N-terminal domain. This fragment is capable of forming pores in the cell membrane, leading to the leakage of cellular contents, and serves as a key effector molecule in pyroptosis (Burdette et al., 2021). WB detection revealed elevated expression levels of NLRP3, cleaved-caspase1, ASC, and GSDMD-N in the injured area of CSR rats compared to the Sham group (all *p* < 0.001); whereas NWM treatment reduced the expression levels of NLRP3, cleaved-caspase1, ASC, and GSDMD-N in the injured area of CSR rats (all *p* < 0.001) (Figures 4C,D). Using Annexin V + PE and 7-AAD double staining to measure pyroptosis levels, it was found that the rate of pyroptotic cells (Annexin V^+^/7-AAD^+^) in the injured area of the CSR group was higher than that in the Sham group (*p* < 0.001); whereas NWM treatment reduced pyroptotic cells in the injured area of CSR rats (*p* < 0.001) (Figures 4E,F). Concurrently, ELISA detection also confirmed that NWM significantly lowered IL-18 levels (a marker of pyrolysis) in CSR rats (Figure 4G).

**Figure 5:**
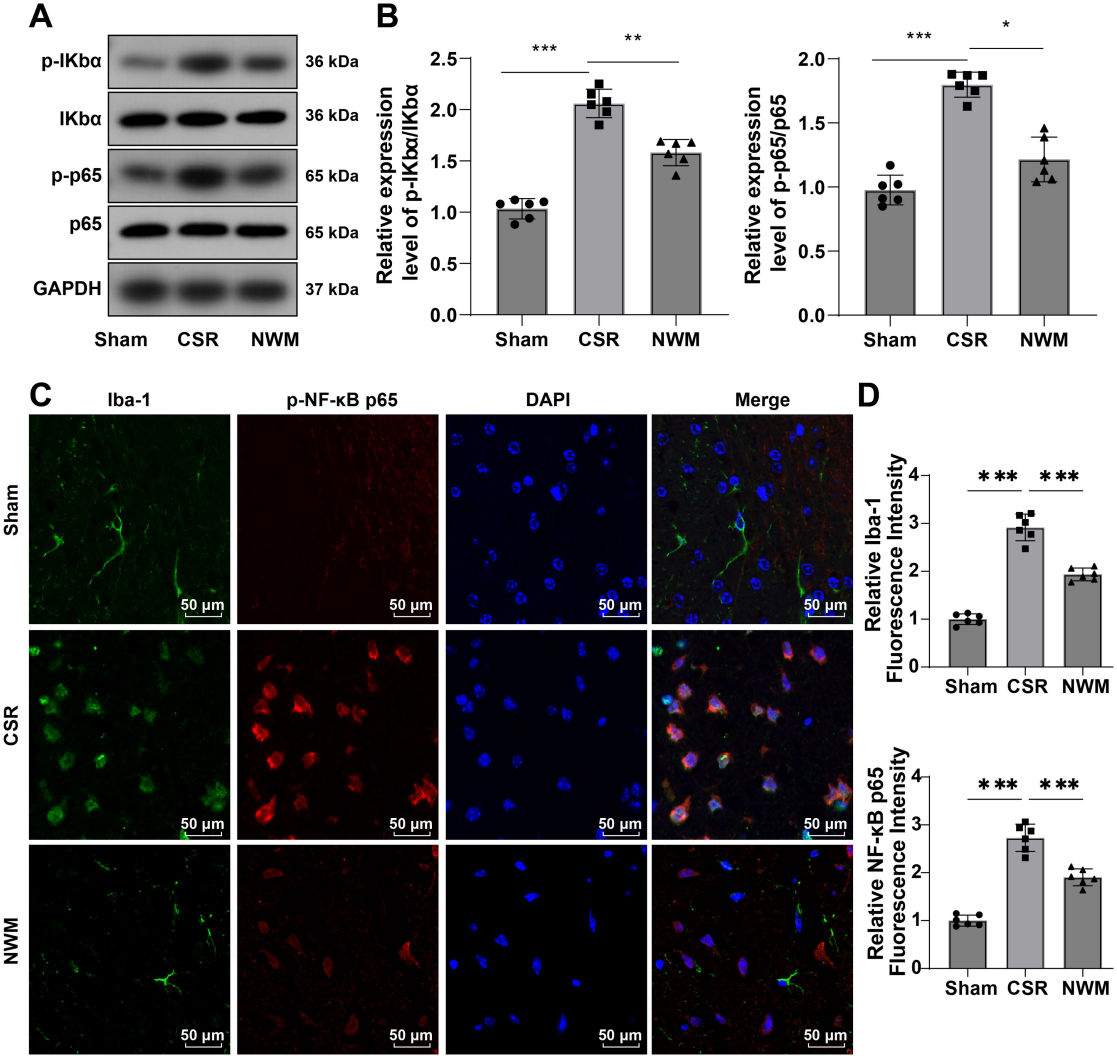
Inhibition of the NF-κB pathway activation in CSR rats by NWM. **(A,B)** Representative immunoblot images and quantitative analysis of p-IKbα, IKbα, p-p65, and p65 protein expression detected by WB; **(C,D)** Expression and quantitative analysis of Iba1 and phosphat-p65 detected by immunofluorescence double staining. *n* = 6. Data were presented as mean ± standard deviation, with multiple group comparisons performed using one-way ANOVA and post-hoc Tukey’s multiple comparison test. * *p* < 0.05, ** *p* < 0.01, *** *p* < 0.001.

As a core regulatory pathway in the inflammatory response, NF-κB activation not only drives the transcription of inflammatory cytokines but also promotes NLRP3 inflammasome activation (Long et al., 2023; Chai et al., 2022). Therefore, the phosphorylation levels of key molecules in the NF-κB signaling pathway were further examined. WB analysis revealed significantly elevated expression of p-IκBα and p-p65 in CSR rats, whereas NWM treatment markedly reduced the phosphorylation levels of both molecules in CSR rats (*p* < 0.05) (Figures 5A,B). Given the pivotal role of microglia in neuroinflammation, immunofluorescence double staining was employed to observe the colocalization of Iba1 and p-p65 (Wan et al., 2019). Results revealed higher co-expression levels of Iba1 and p-p65 in the lesion area of CSR rats compared to Sham rats. Following NWM treatment, co-expression levels of Iba1 and p-p65 decreased in the lesion area of CSR rats (Figures 5C,D). These findings indicate that NWM suppresses NF-κB activation and inhibits NLRP3 inflammasome-induced pyroptosis, suggesting this may represent a key molecular mechanism underlying its amelioration of CSR-induced inflammatory injury.

### 3.4 NWM regulates mitochondrial morphology and intracellular ROS in CSR rats, inhibiting mitochondrial dysfunction

**Figure 6:**
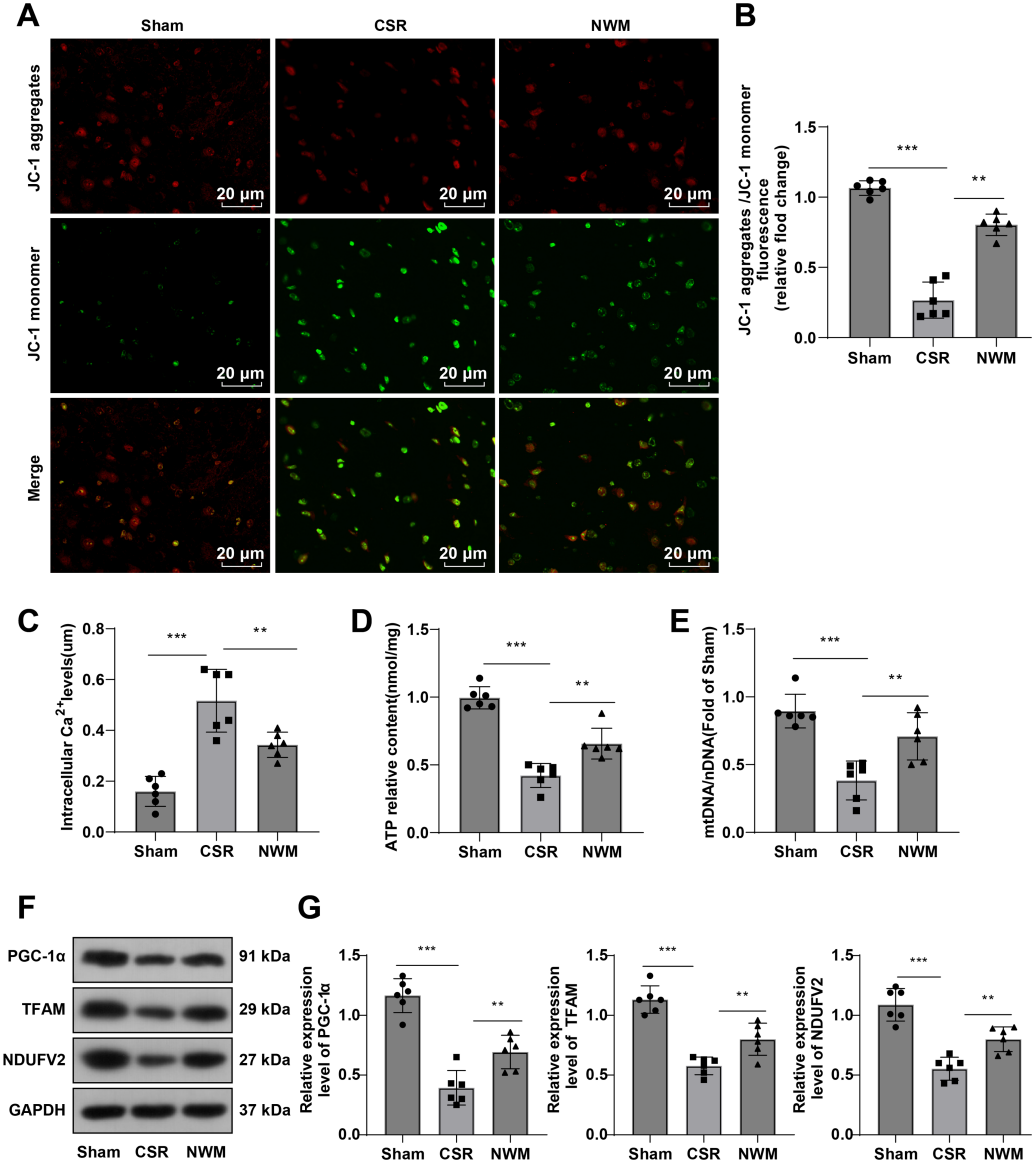
Regulation of mitochondrial morphology and inhibition of mitochondrial dysfunction in CSR rats mediated by NWM. **(A,B)** Mitochondrial membrane potential levels detected by the JC-1 assay kit; **(C)** Ca^2+^ levels detected by the Fluo-3/AM fluorescence probe; **(D)** ATP levels in mitochondria detected using the kit; **(E)** mtDNA analyzed by RT-qPCR; **(F,G)** Representative immunoblot images and quantitative analysis of PGC-1α, TFAM, and NDUFV2 protein expression detected by WB. *n* = 6. Data were presented as mean ± standard deviation, with multiple group comparisons performed using one-way ANOVA and post-hoc Tukey’s multiple comparison test. ** *p* < 0.01, *** *p* < 0.001.

Activation of the NF-*κ*B pathway is closely associated with ROS levels, and mitochondria, as the primary intracellular source of ROS, may play a critical role in this process (Li et al., 2022; Xu et al., 2024). To validate this hypothesis, the effects of NWM on mitochondrial function in CSR rats were further investigated. JC-1 assay kit was used to detect mitochondrial membrane potential levels. MMP in the CSR group was lower than that in the Sham group (Figures 6A,B), while MMP significantly recovered after NWM intervention. Simultaneously, multiple indicators of mitochondrial function were assessed. The CSR group exhibited elevated cytoplasmic Ca^2+^ levels, reduced ATP production, and decreased mitochondrial DNA copy numbers in spinal cord tissue. NWM treatment effectively improved these CSR-induced alterations (*p* < 0.001) (Figures 6C–E). Furthermore, WB detection of key molecules in mtDNA replication and mitochondrial transcription, PGC-1*α*, TFAM, and NDUFV2, revealed significantly lower levels in the injured tissue of CSR rats compared to Sham rats (all *p* < 0.001) (Figures 6F,G). Following NWM treatment, PGC-1*α*, TFAM, and NDUFV2 levels significantly recovered in CSR rats (all *p* < 0.01) (Figures 6F,G). These findings indicate that NWM improves mitochondrial function and maintains mitochondrial homeostasis in CSR rats.

**Figure 7:**
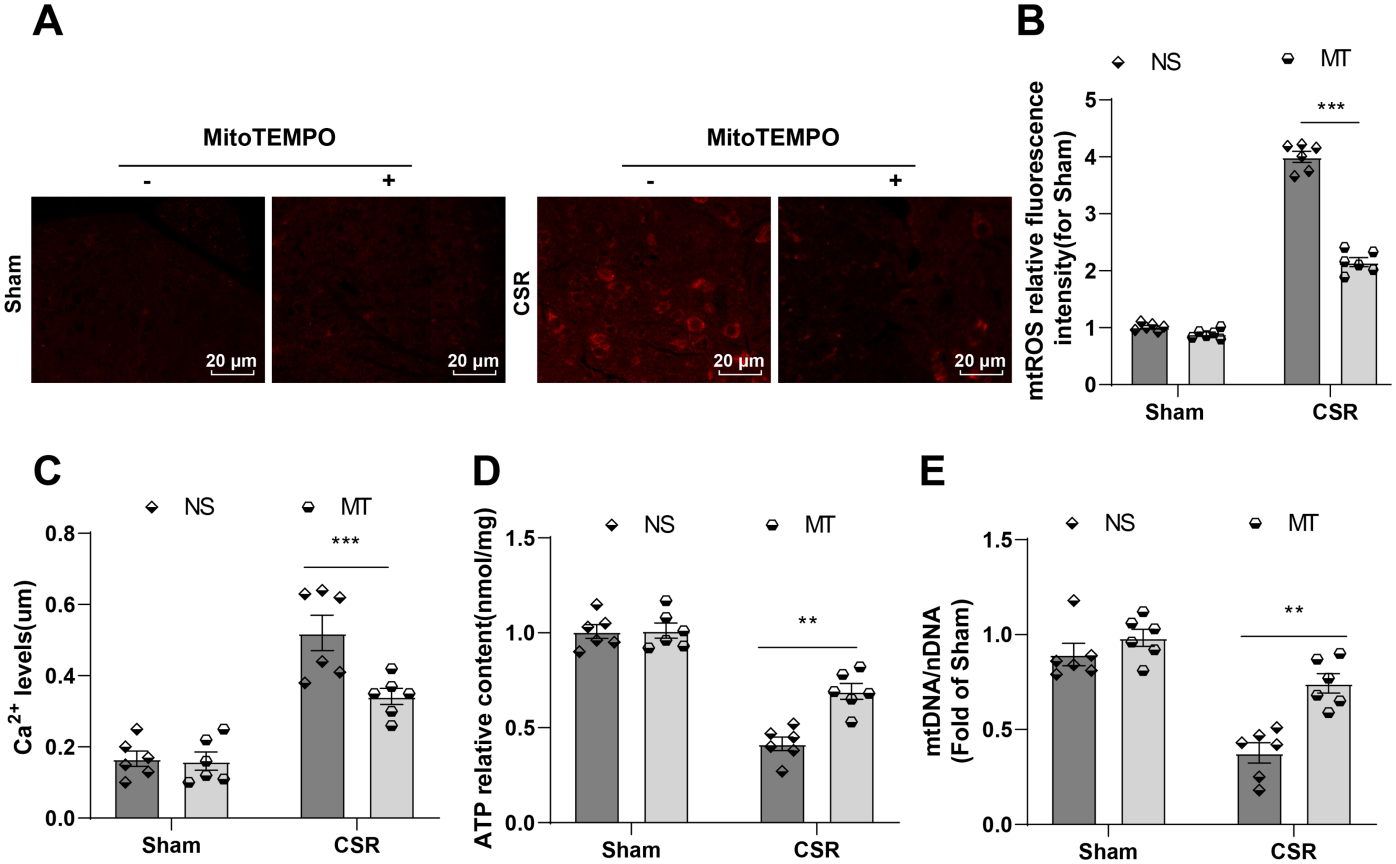
Inhibition of mtROS on the improvement of mitochondrial function in CSR rats. **(A,B)** MitoSOX fluorescence (red) for assessing mtROS levels; **(C)** Ca^2+^ levels detected by the Fluo-3/AM fluorescence probe; **(D)** ATP levels in mitochondria detected using the kit; **(E)** mtDNA analyzed by RT-qPCR. *n* = 6. Data were presented as mean ± standard deviation, with multiple group comparisons performed using one-way ANOVA and post-hoc Tukey’s multiple comparison test. *** *p* < 0.001.

Mitochondrial dysfunction leading to excessive mtROS can trigger pyroptosis, thereby regulating caspase-1/GSDMD-dependent pyroptosis (Wan et al., 2019). Therefore, the intrinsic relationship between mitochondrial function and pyroptosis was further investigated. Rats with CSR were treated with the mitochondria-specific antioxidant MitoTEMPO. MitoSOX fluorescence was used to validate the critical role of mtROS in the pyroptosis pathway. It was found that mtROS in the injured tissue of CSR rats was higher than in the Sham group (Figures 7A,B). Subsequently, sham-operated rats or CSR rats were treated with the MitoTEMPO. It was found that compared to the NS group, mtROS in the injured tissue of the MitoTEMPO group rats was suppressed (*p* < 0.01) (Figures 7A,B). Consistent with this, MitoTEMPO treatment also significantly reduced cytoplasmic Ca^2+^ levels in CSR rats while enhancing mitochondrial ATP production and mtDNA copy number (*p* < 0.01) (Figures 7C–E). These results indicate that NWM may suppress NLRP3 inflammasome activation and neuronal pyroptosis by improving mitochondrial function and reducing mtROS production, providing crucial experimental evidence for understanding its mechanism in treating CSR.

### 3.5 NWM reduces VAS and NDI scores and mitigates inflammatory responses in CSR patients

**Figure 8:**
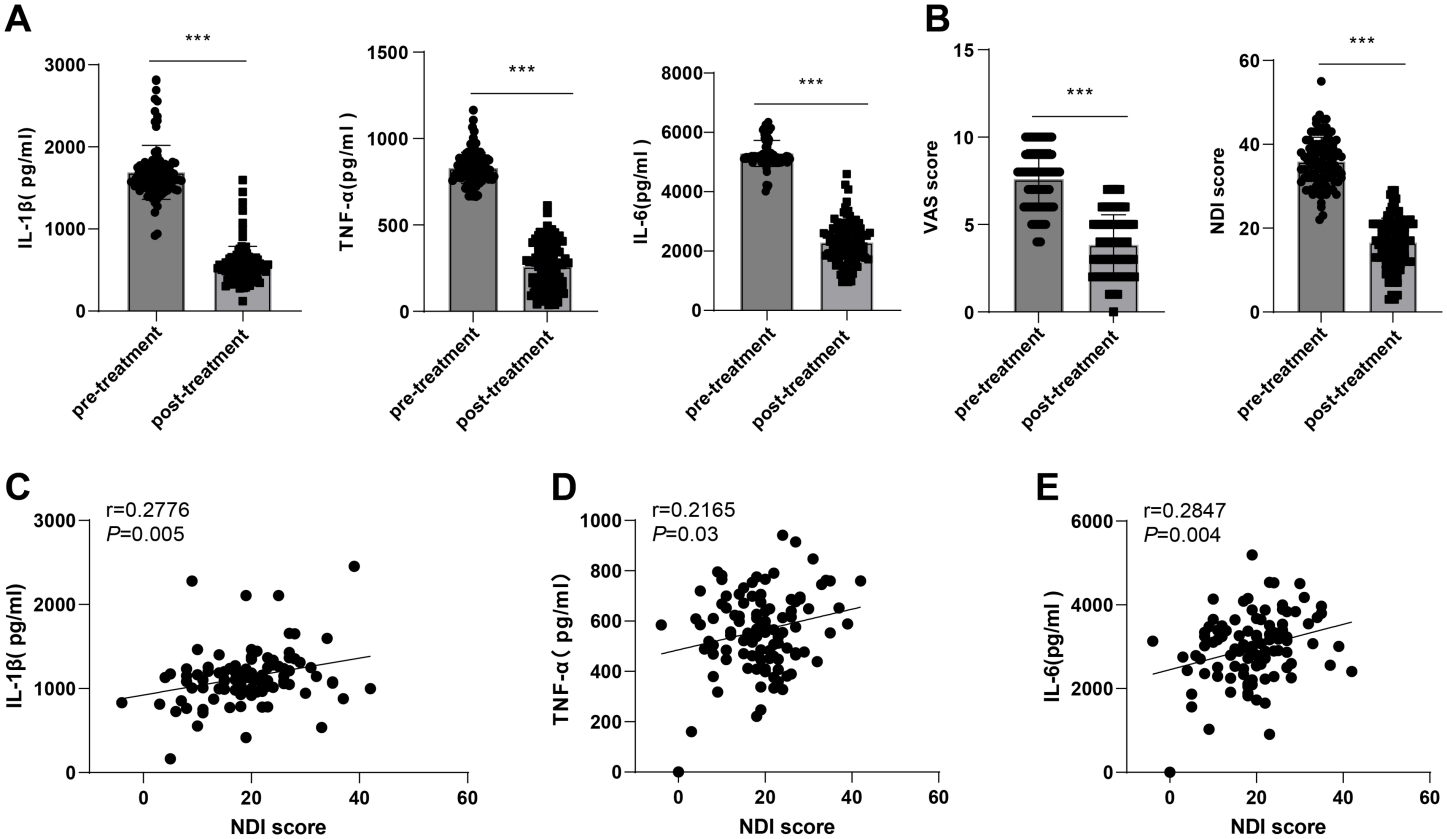
Reduction of VAS, NDI scores, and inflammatory responses in CSR patients by NWM. **(A)** ELISA detection of inflammatory factors IL-1β, TNF-α, and IL-6 levels in serum of CSR patients before and after NWM; **(B)** VAS and NDI scores of CSR patients before and after NWM; **(C–E)** Correlation analysis of the difference in NDI scores before and after NWM treatment in patients with CSR and the difference in serum levels of the inflammatory cytokines IL-1β, TNF-α, and IL-6. Independent samples t-tests were used for comparisons between the two groups; Pearson correlation analysis was used for the correlation analysis, with *r* as the correlation coefficient, ** *p* < 0.01, *** *p* < 0.001.

A clinical study was conducted to analyze the effects of NWM on clinical symptoms and systemic inflammation levels in CSR patients. ELISA was carried out to determine the levels of inflammatory factors IL-1β, TNF-*α*, and IL-6 in the serum of 100 CSR patients before and after NWM. It was found that NWM significantly reduced the levels of inflammatory factors IL-1β, TNF-α, and IL-6 in the serum of CSR patients (*p* < 0.001) (Figure 8A). VAS and NDI scores of CSR patients were significantly reduced after treatment (*p* < 0.001) (Figure 8B). To further clarify the intrinsic relationship between the reduction in inflammatory markers and the improvement in clinical symptoms, the improvement in NDI scores and levels of the three inflammatory markers before and after treatment were calculated for each patient, and a Pearson correlation analysis was performed. The results showed that the improvement in NDI scores was significantly positively correlated with the reductions in IL-1β, TNF-α, and IL-6 (*r* = 0.2776, *p* = 0.005; *r* = 0.2165, *p* = 0.03; *r* = 0.2847, *p* = 0.004) (Figures 8C–E). These results suggest that NWM treatment not only reduces systemic inflammation and improves clinical symptoms in patients with CSR, but also that the reduction in inflammation is closely associated with the alleviation of clinical symptoms, providing clinical support for the mechanisms identified in animal studies.

## 4 Discussion

Dysregulated mitochondrial dynamics are increasingly recognized as a key factor in the etiology of a spectrum of neurodegenerative diseases, suggesting that the modulation of these dynamics can potentially offer significant therapeutic advantages in the clinical management of CSR (Zhou et al., 2020). Extending beyond mere structural anomalies, the pathogenic mechanisms encompass a complex interplay of mitochondrial dysfunction, heightened oxidative stress, and inflammatory responses, which collectively activate cell death cascades, including apoptosis and pyroptosis (Kong et al., 2024). Notably, neuronal pyroptosis has been implicated in the pathogenesis and progression of spinal cord injuries, representing a severe form of neurological impairment (Yuan et al., 2022). Drawing from these findings, we posit that an in-depth exploration into mitochondrial preservation strategies and the role of neuronal pyroptosis is imperative for a more comprehensive understanding of their involvement in the initiation, advancement, and outcome of CSR. Empirical evidence has demonstrated that acupuncture can mitigate oxidative stress, curtail iron deposition, and ameliorate mitochondrial damage (Hao et al., 2024). Furthermore, acupuncture has been shown to suppress hippocampal neuronal pyroptosis in Alzheimer’s disease, a prevalent neurodegenerative condition (Zhu et al., 2024a). In the context of this study, we endeavored to scrutinize the therapeutic impacts of NWM within a rat model of CSR, delving into the underlying mechanisms that might account for its efficacy.

Following the establishment of a CSR rat model utilizing the fish wire extrusion method, a technique widely acknowledged in the field (Su et al., 2023), our investigation revealed that NWM significantly elevated PPT and MPT in CSR rats. It is noteworthy that PPT, a parameter frequently employed in behavioral research, is inversely correlated with pain intensity and disability, with higher PPT values reflecting reduced pain and disability levels (Winarno et al., 2025). Furthermore, gait, often considered a largely automated motor task requiring minimal higher-level cognitive involvement (Yogev-Seligmann et al., 2008), was markedly improved in the NWM group, as indicated by significantly lower gait scores, thereby demonstrating enhanced motor function. SP, PGE2, and NPY are recognized as key stressors in pain pathways (Zhu et al., 2023). Notably, SP facilitates glutamate release and amplifies nociceptive signals, thereby participating in the genesis and perpetuation of pain (He et al., 2024). Our study observed a reduction in SP levels following NWM treatment. Beyond motor function, we delved into the impacts of NWM on inflammatory responses and neuronal damage, recognizing that neuronal activation and the secretion of inflammatory mediators are instrumental in the development of neuropathic pain (Moalem and Tracey, 2006). The expression of proinflammatory cytokines, such as TNF-*α*, has been well-documented in modulating precise cellular events, including the activation of astrocytes (Paterniti et al., 2014). In line with our findings, a previous study has reported significant reductions in serum IL-6 and TNF-α levels in the observation group post-NWM treatment (Lu et al., 2021). Moreover, neuronal damage or apoptosis plays a determinant role in the degree of neurological deficit in cervical spondylotic myelopathy to a certain extent (Karadimas et al., 2013). Our TUNEL staining results demonstrated a reduction in TUNEL-positive neurons following NWM treatment, suggesting a protective effect against neuronal cell death. In neuropathic pain conditions such as CSR, the expression of synaptic proteins such as α-syn, synapsin, and PSD-95 is abnormally elevated, reflecting pathological synaptic remodeling and the development of central sensitization. This abnormally enhanced synaptic transmission is one of the core mechanisms underlying pain hypersensitivity (Liang et al., 2025; Wang et al., 2020b; Zhang et al., 2019). Therefore, the elevated levels of these proteins in CSR rats represent pathological changes, and the subsequent decline in their expression following needle-warming moxibustion intervention should be interpreted as a reversal or correction of the pathological synaptic remodeling induced by CSR. This restores abnormal synaptic structure and function to a normal state, thereby alleviating central sensitization and exerting analgesic effects.

Expanding on the therapeutic mechanisms of NWM in CSR, we delved into the role of NWM in curbing ROS accumulation, a strategy known to ameliorate neuronal apoptosis (Zhu et al., 2024b). Given that the inhibition of the NF-κB pathway can mitigate ROS levels within inflammatory environments (Xie et al., 2024), our investigation centered on this pathway. Consistent with our observations, a preceding study has documented the reduction of NF-κB, following the application of acupuncture and moxibustion (Yang et al., 2024b). Concurrently, NF-κB stands as a principal pathway in the inflammatory response; its activation and subsequent nuclear translocation are not only instrumental in the transcription of genes encoding cytokines and chemokines but also influence the activation of the downstream NLRP3 pathway (Long et al., 2023). Interestingly, electroacupuncture, which enhances gastrointestinal motility, may exert its effects through the inhibition of NLRP3 inflammasome and pyroptosis mediated by the NLRP3/caspase-1/GSDMD pathway (Huang et al., 2025). Our study demonstrated that NWM suppressed the activation of NLRP3 inflammasomes and the subsequent pyroptosis triggered by NF-κB inhibition. In summary, within the CSR model, nerve root compression induces mitochondrial dysfunction, leading to excessive mtROS production. Excessive ROS directly activates the NF-κB signaling pathway, promoting inflammatory cytokine release and NLRP3 inflammasome activation, while simultaneously exacerbating mitochondrial dysfunction through oxidative damage, creating a vicious cycle. Activated NLRP3 induces neuronal pyroptosis via the caspase-1/GSDMD pathway, ultimately leading to sustained nerve damage and pain. The therapeutic effect of NWM is achieved precisely by improving mitochondrial function and interrupting this vicious cycle.

This study has limitations. First, only male rats were used in this study. Considering that sex dimorphism has been widely demonstrated in neuroinflammation and pain processing, future research should incorporate female animal models and conduct clinical sex-stratified analyses to systematically evaluate the impact of sex factors on the efficacy of NWM for CSR. Second, this study elucidated the mechanism by which NWM improves CSR through regulating the NF-κB/ROS/NLRP3 pathway and mitochondrial function. However, the upstream regulatory molecules in this pathway and the direct targets of NWM remain incompletely characterized. Furthermore, the specific contribution of glial-neuronal interactions to CSR pathogenesis and the differential regulatory effects of NWM on distinct cell types warrant further investigation. Third, the clinical sample size in this study was relatively limited and derived from a single center, potentially introducing selection bias. Although significant reductions in patients’ VAS, NDI, and inflammatory cytokine levels were observed following NWM treatment, the absence of long-term follow-up data precludes assessment of its long-term efficacy and recurrence rates. Fourth, since all experimental samples have already been used for the planned assays and cannot be replenished, double immunostaining with Iba1 and a neuronal marker and low-magnification images are not accessible. Future studies should conduct multicenter, large-sample, randomized controlled trials incorporating long-term follow-up to comprehensively evaluate the clinical value of NWM in treating CSR.

In conclusion, our experimental findings underscore the neuroprotective properties of NWM. We have illustrated that NWM fostered the recovery of motor function and alleviated neuropathic pain in the aftermath of CSR. The potential mechanisms appear to involve the inhibition of NLRP3 inflammasomes and the prevention of pyroptosis induced by NF-κB inhibition. Clinically, these findings suggest that NWM may offer a valuable adjunct or alternative therapeutic strategy in the management of CSR.

## Data Availability

The original contributions presented in the study are included in the article/Supplementary material, further inquiries can be directed to the corresponding author.
