# Supplementary figures and images for: Needle-warming moxibustion alleviates pain in rats with cervical spondylotic radiculopathy by modulating the NF-κB/ROS/NLRP3 pathway
Source: Front Aging Neurosci. 2026 May 28;18:1769489. doi: 10.3389/fnagi.2026.1769489 (PMC13253949; doi:10.3389/fnagi.2026.1769489)

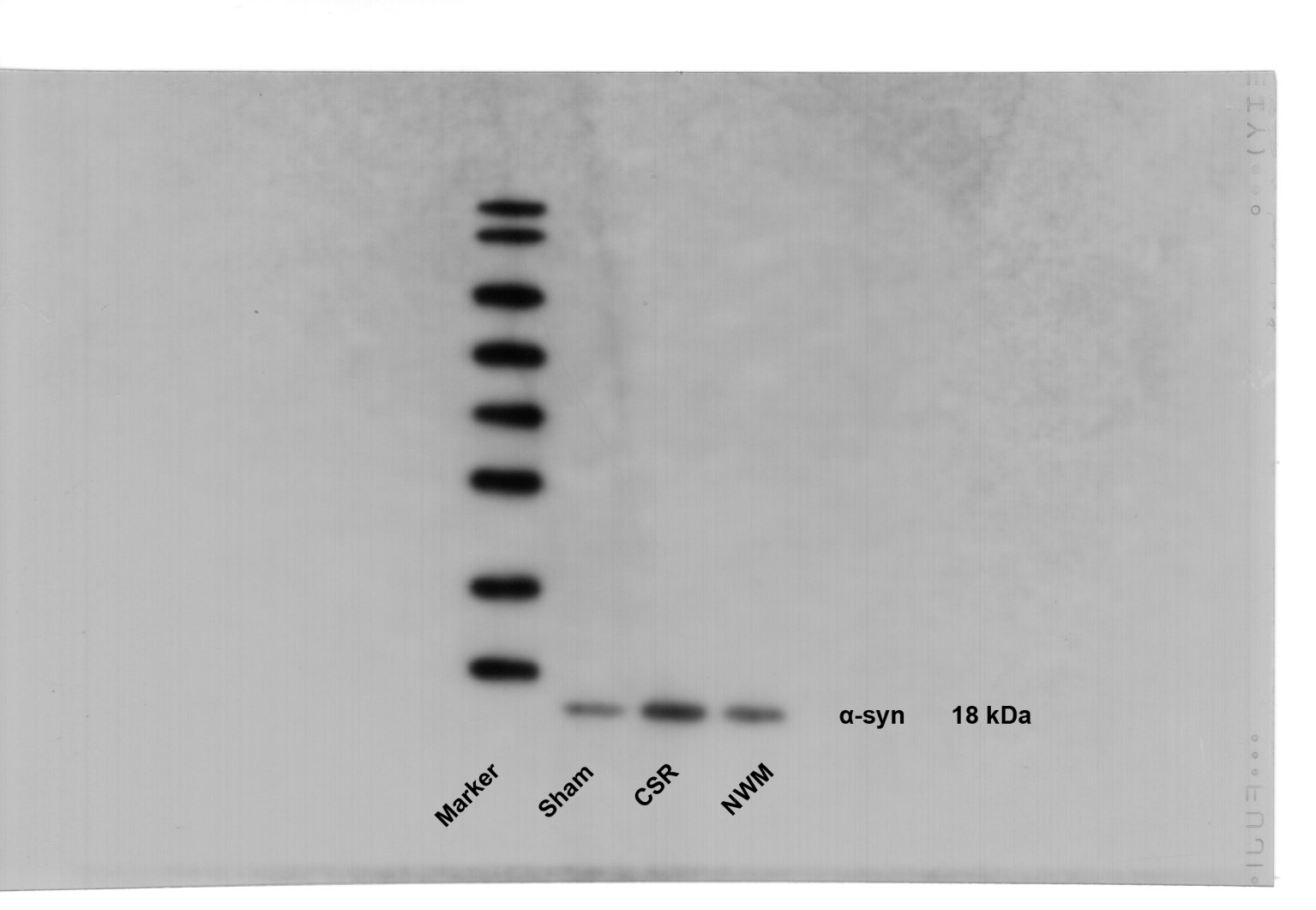

Supplement: Supplementary file 1 [file Data_Sheet_1.ZIP › 3A/1、α-Syn.tif]

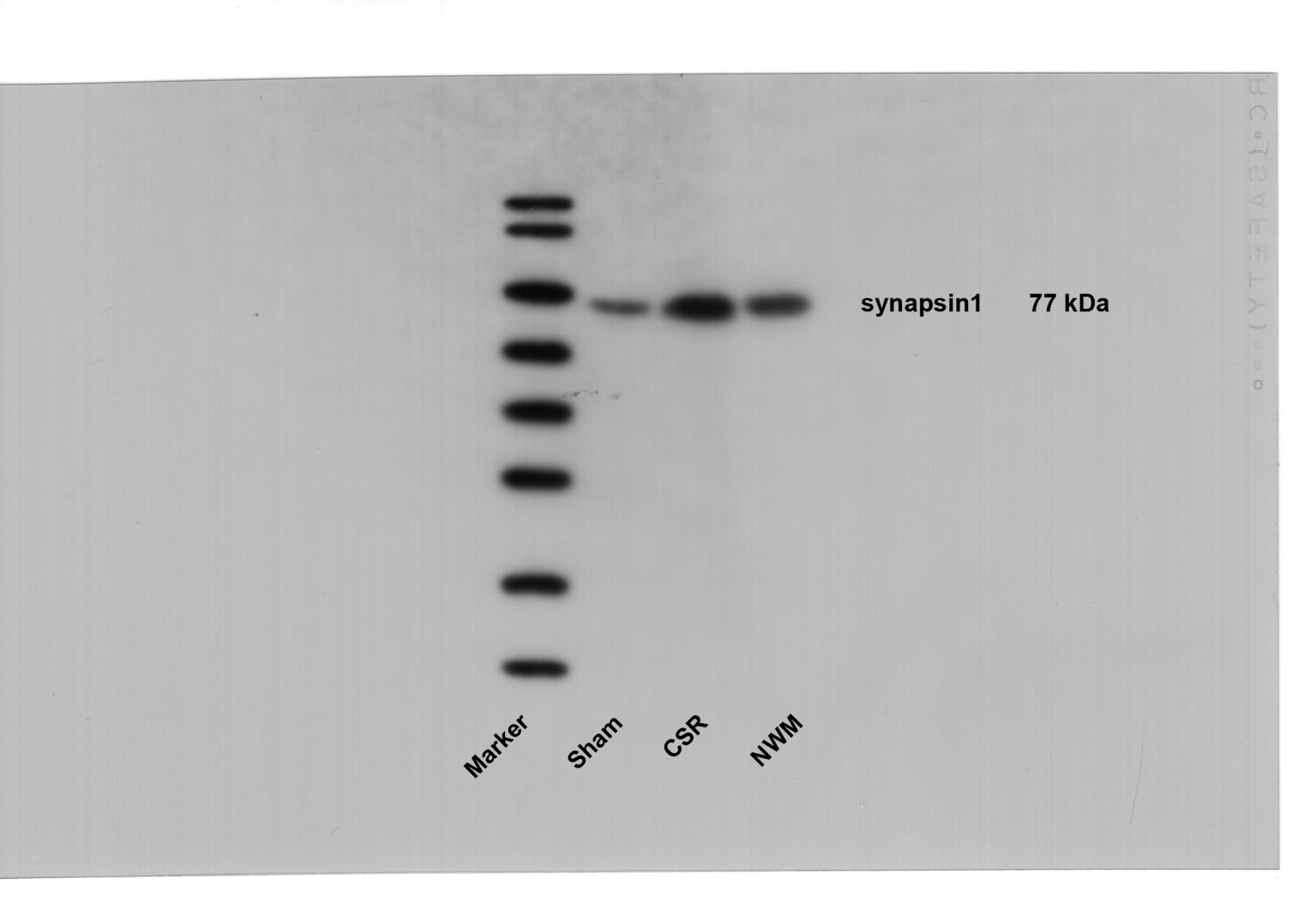

Supplement: Supplementary file 1 [file Data_Sheet_1.ZIP › 3A/2、Synapsin1.tif]

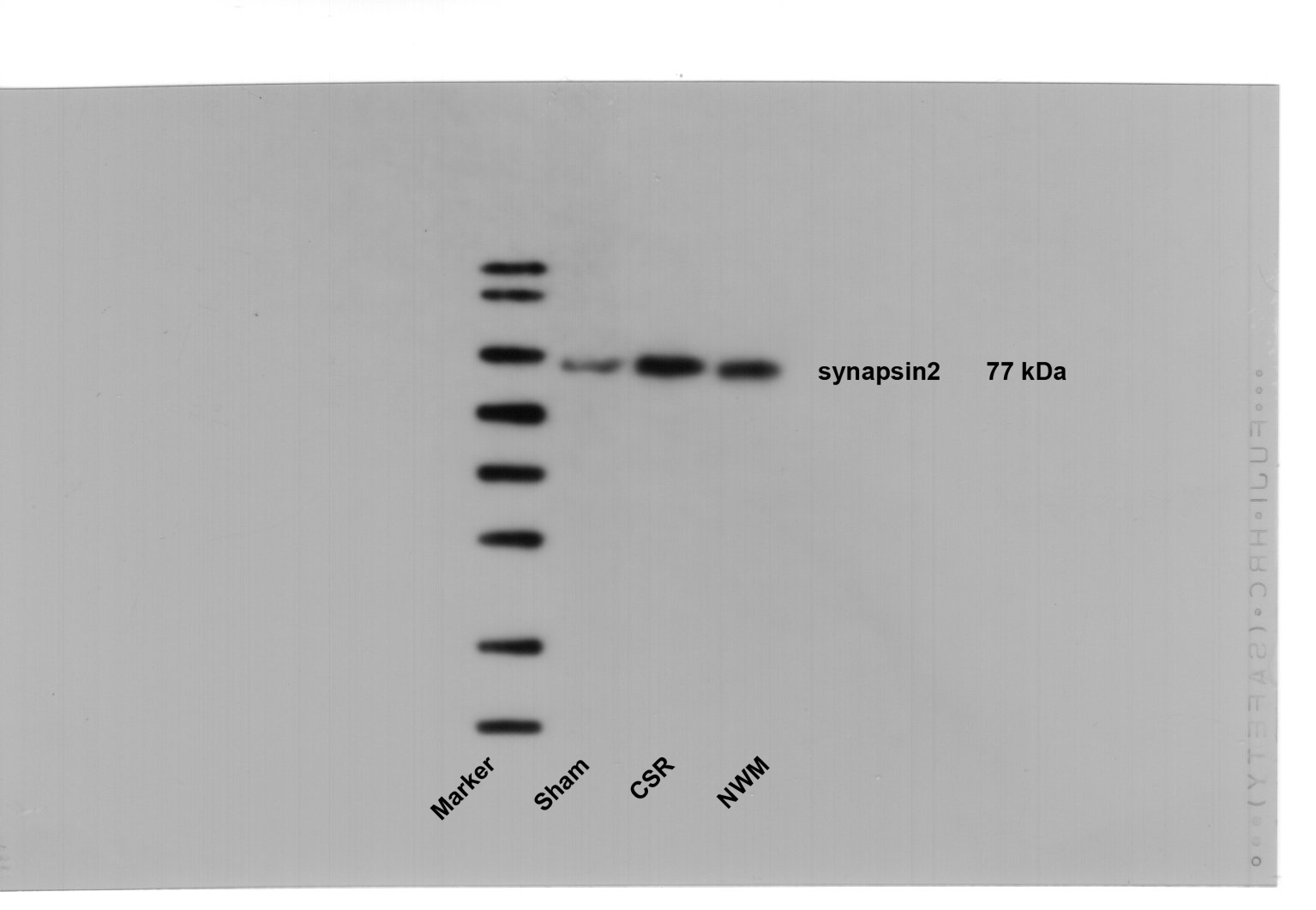

Supplement: Supplementary file 1 [file Data_Sheet_1.ZIP › 3A/3、Synapsin2.tif]

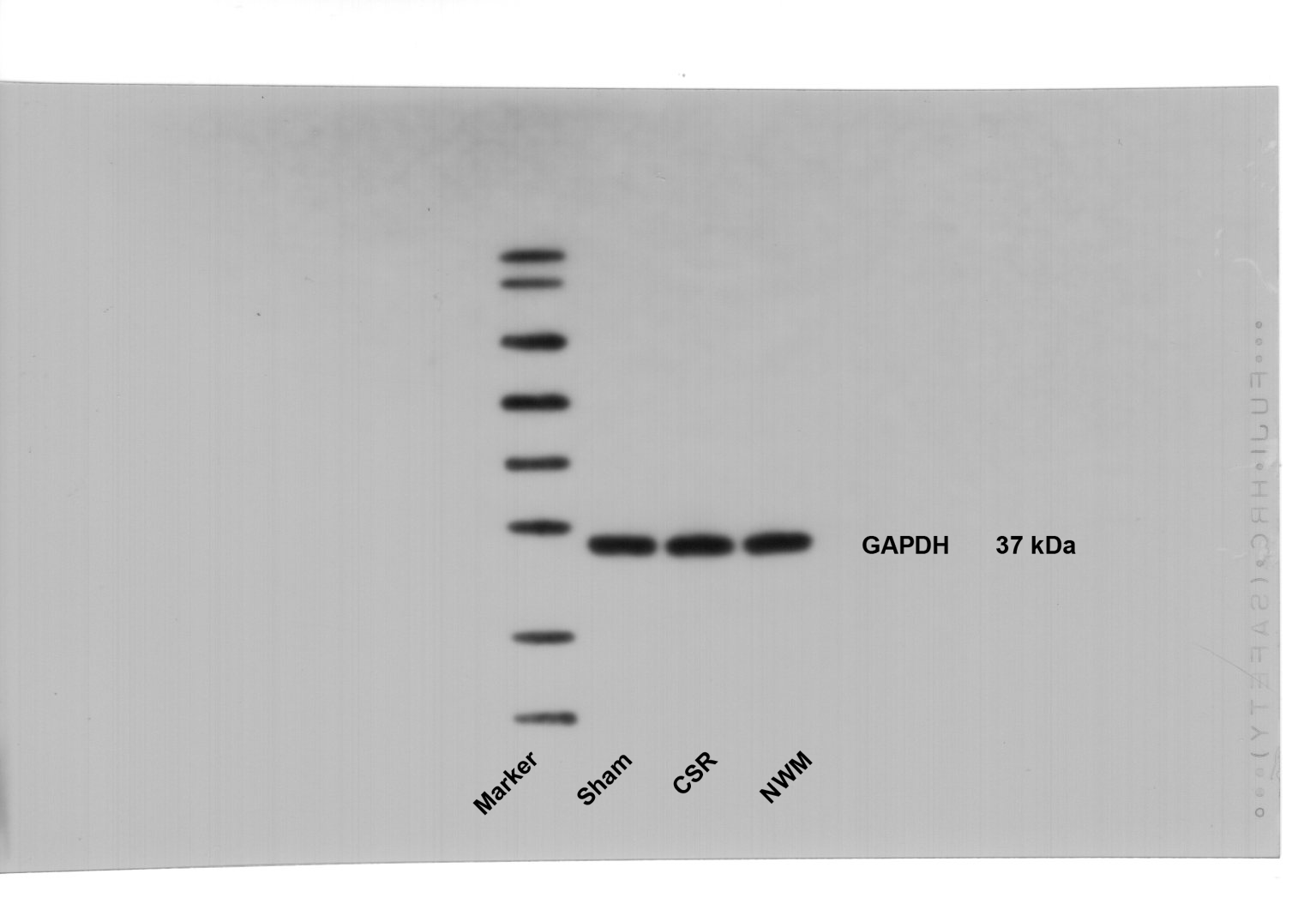

Supplement: Supplementary file 1 [file Data_Sheet_1.ZIP › 3A/4、GAPDH.tif]

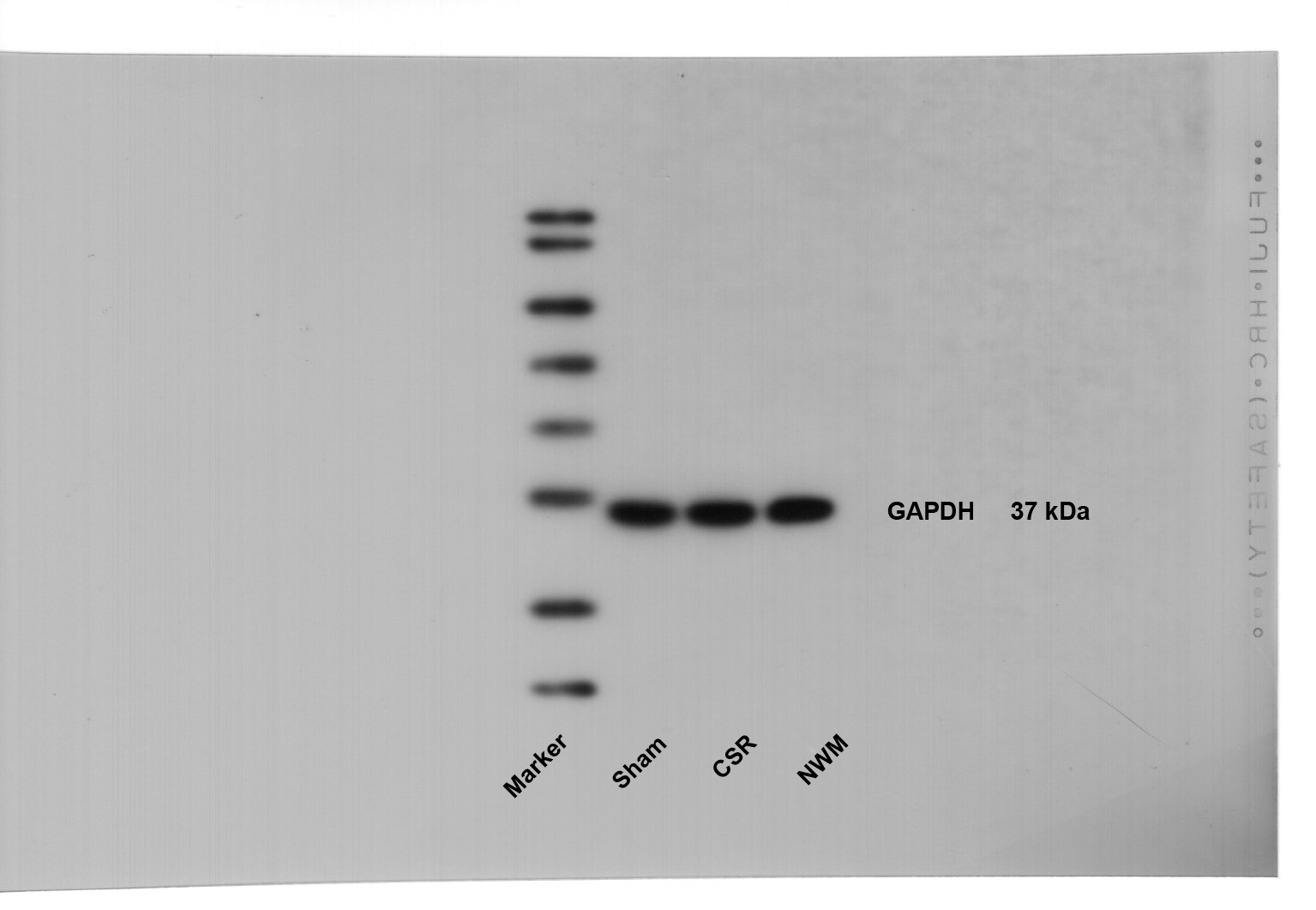

Supplement: Supplementary file 1 [file Data_Sheet_1.ZIP › 4C/10、GAPDH.tif]

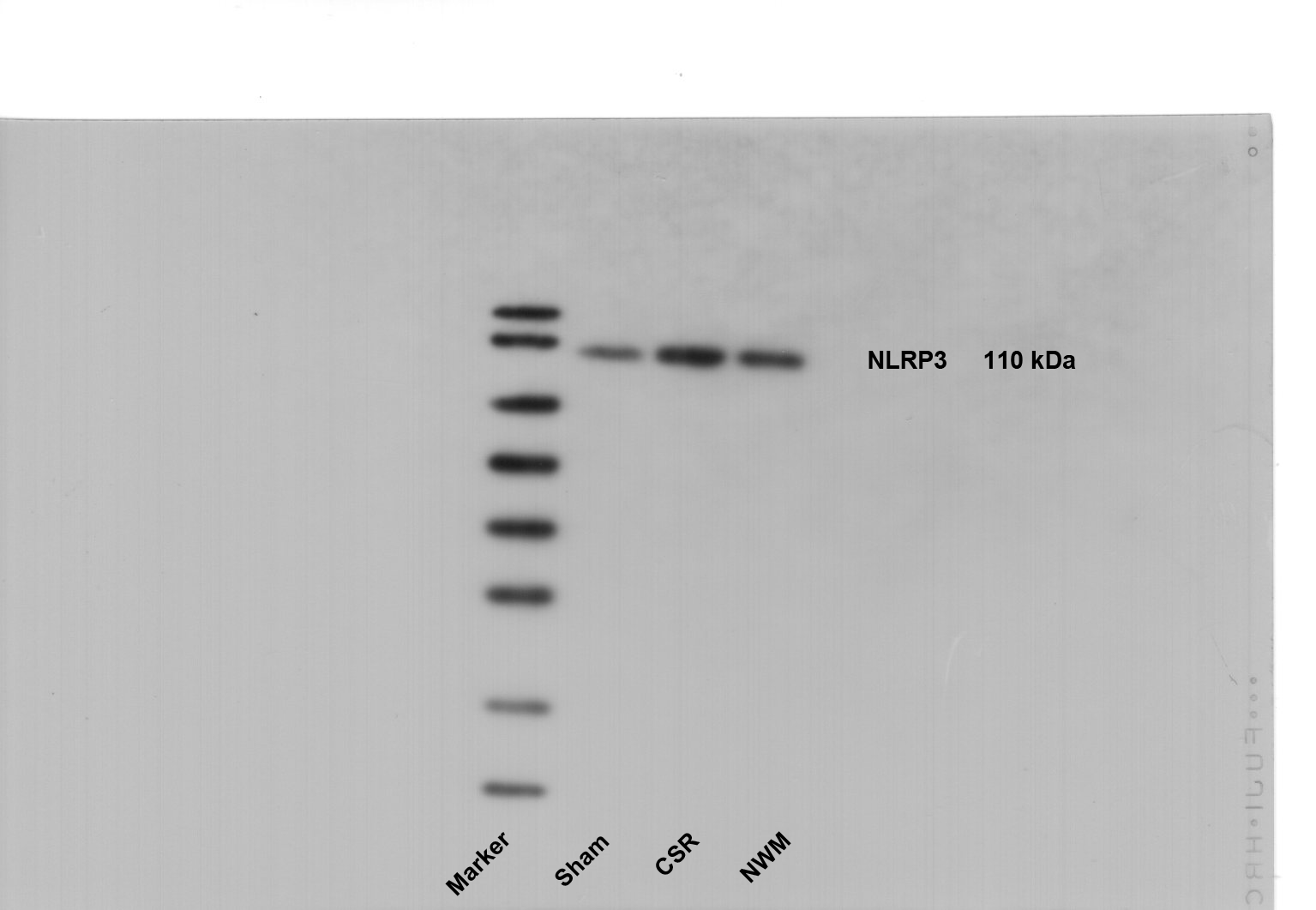

Supplement: Supplementary file 1 [file Data_Sheet_1.ZIP › 4C/5、NLRP3.tif]

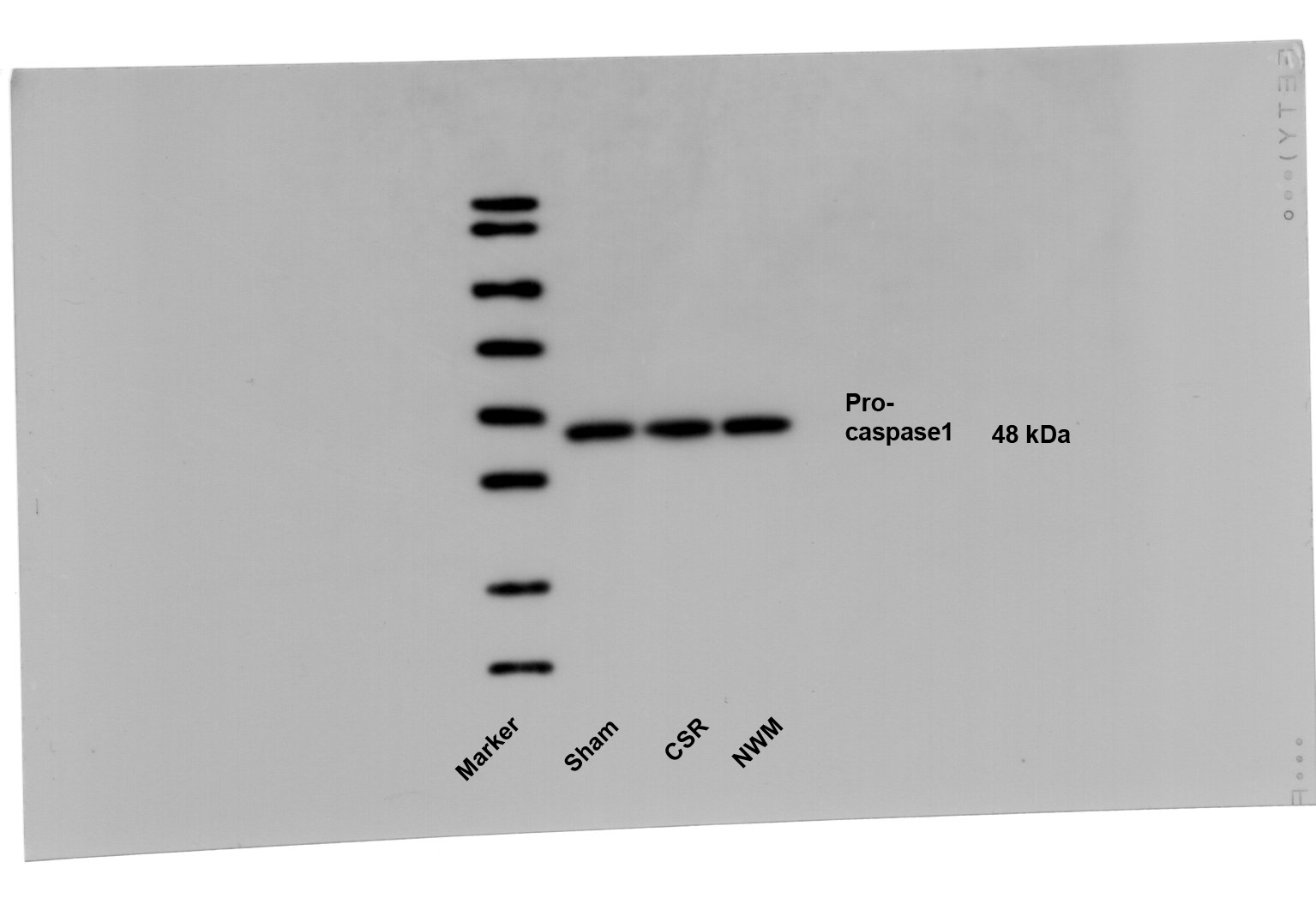

Supplement: Supplementary file 1 [file Data_Sheet_1.ZIP › 4C/6、Pro-Caspase1.tif]

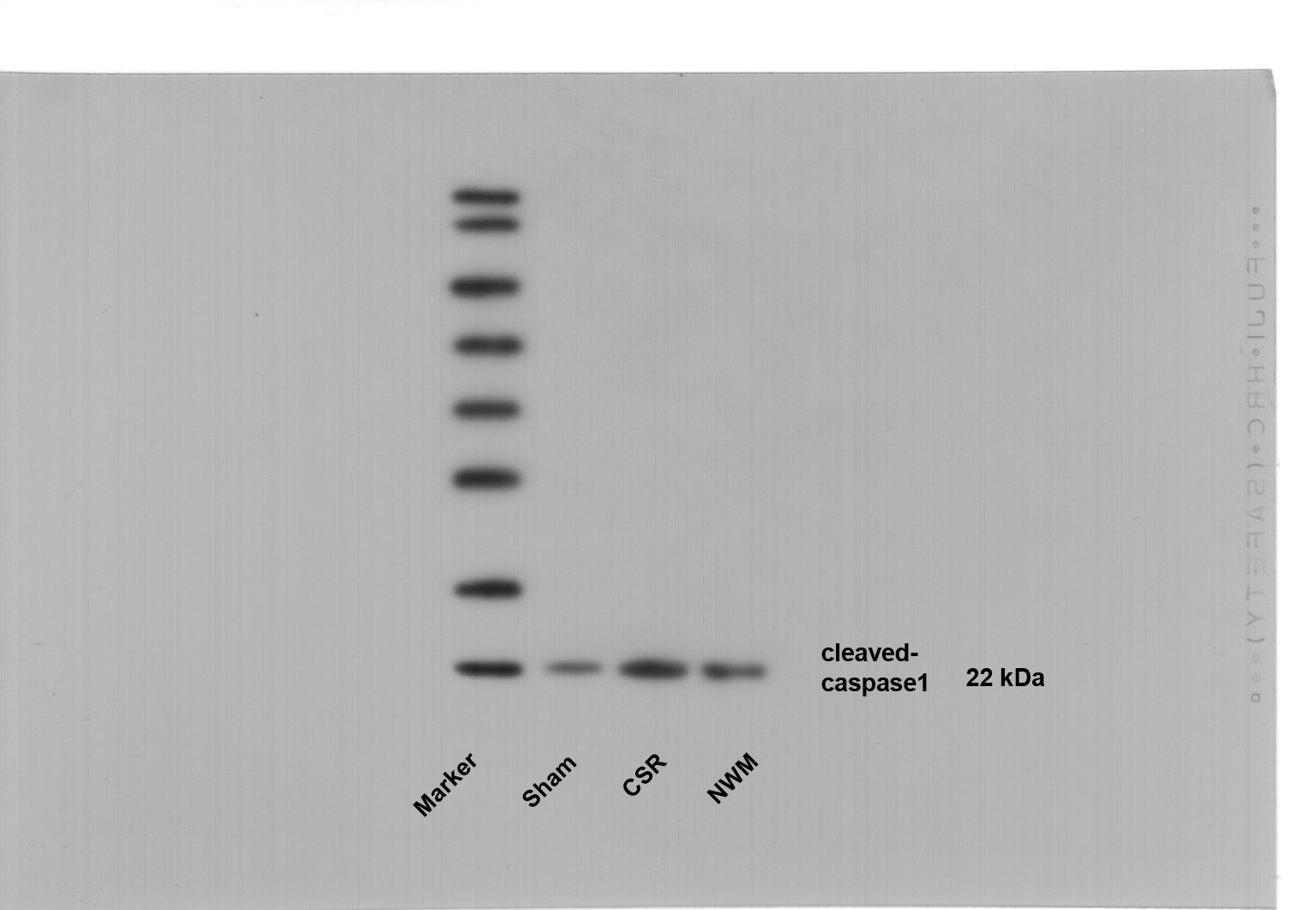

Supplement: Supplementary file 1 [file Data_Sheet_1.ZIP › 4C/7、C-Caspase1.tif]

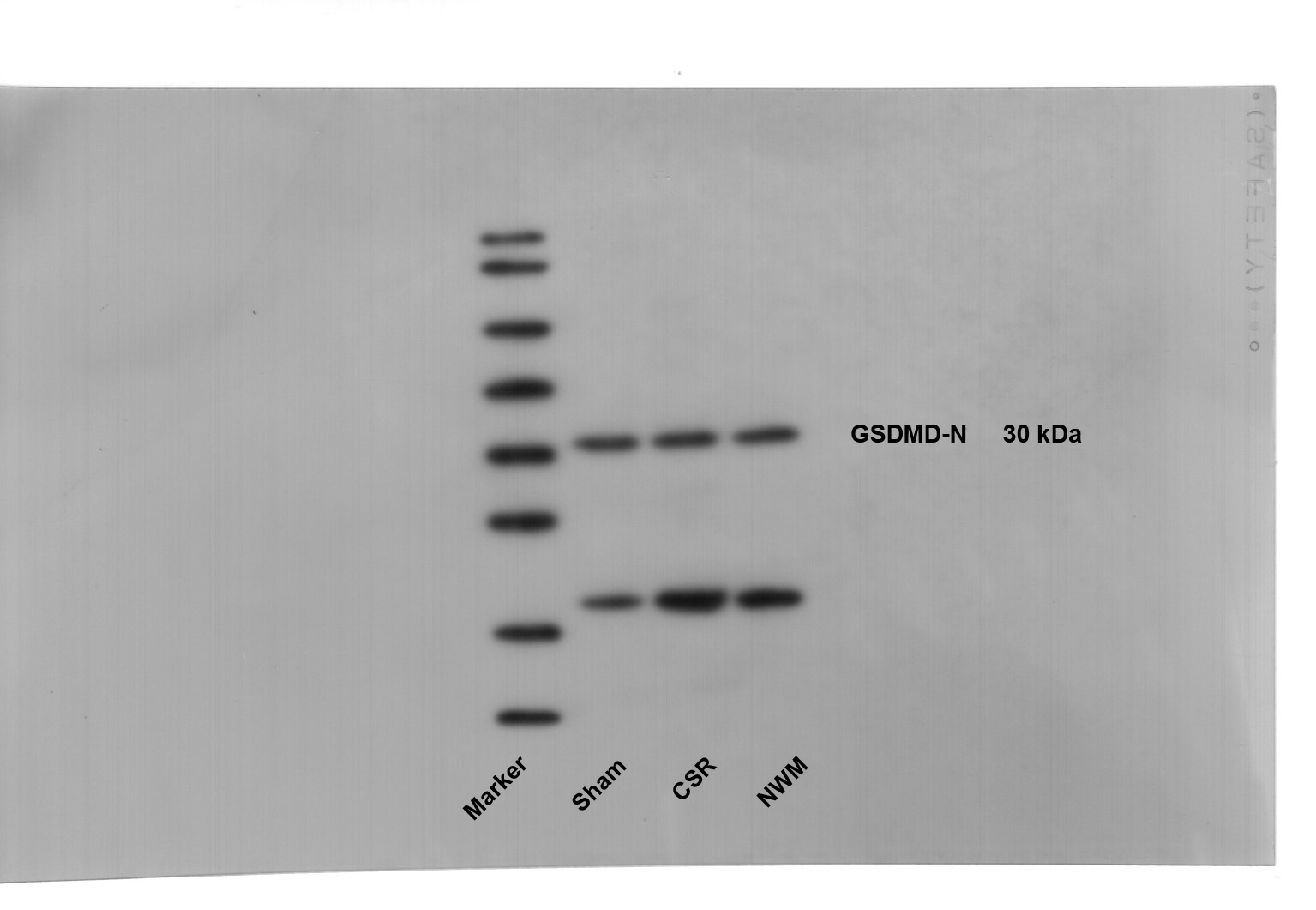

Supplement: Supplementary file 1 [file Data_Sheet_1.ZIP › 4C/8、GSDMD-N.tif]

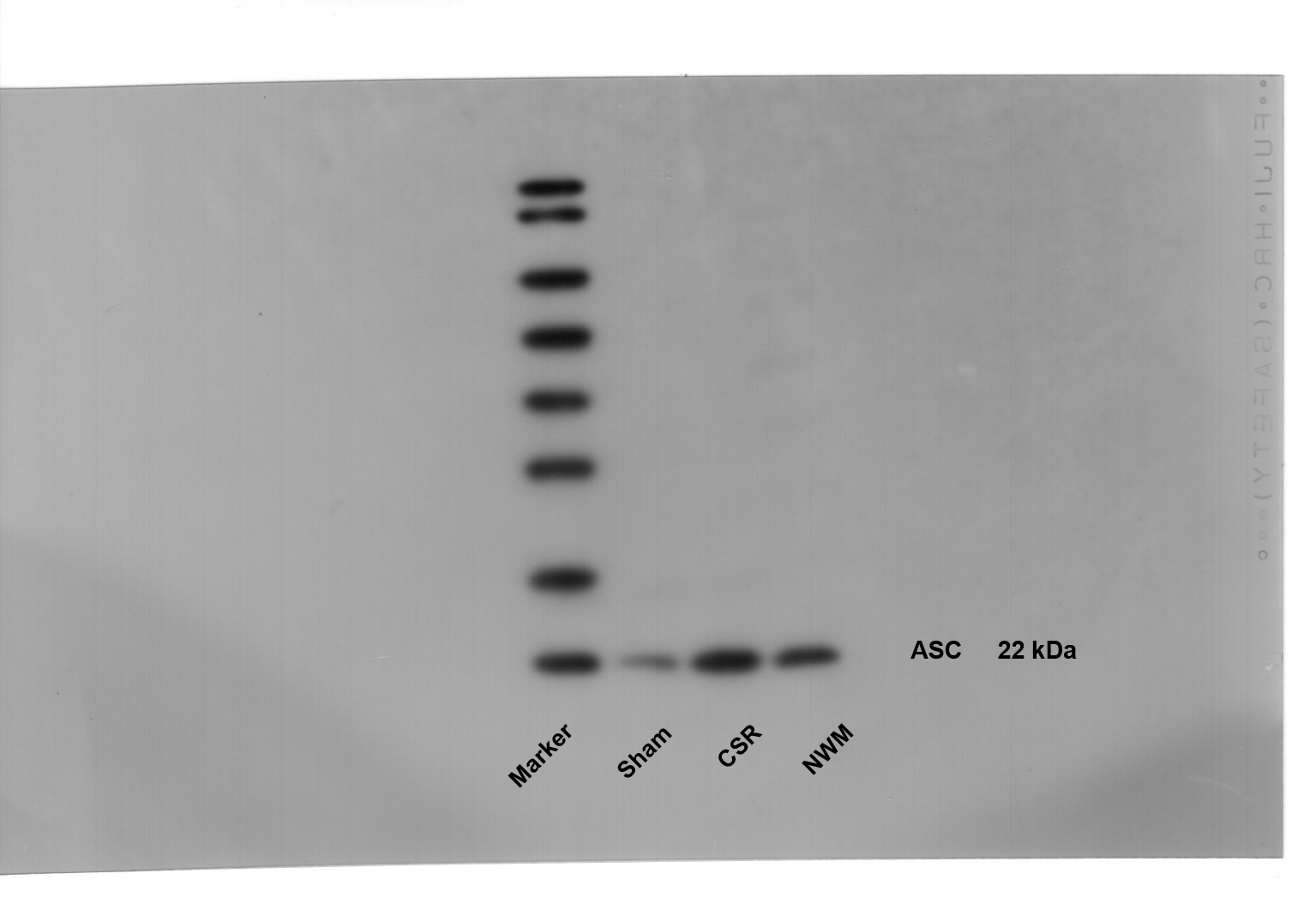

Supplement: Supplementary file 1 [file Data_Sheet_1.ZIP › 4C/9、ASC.tif]

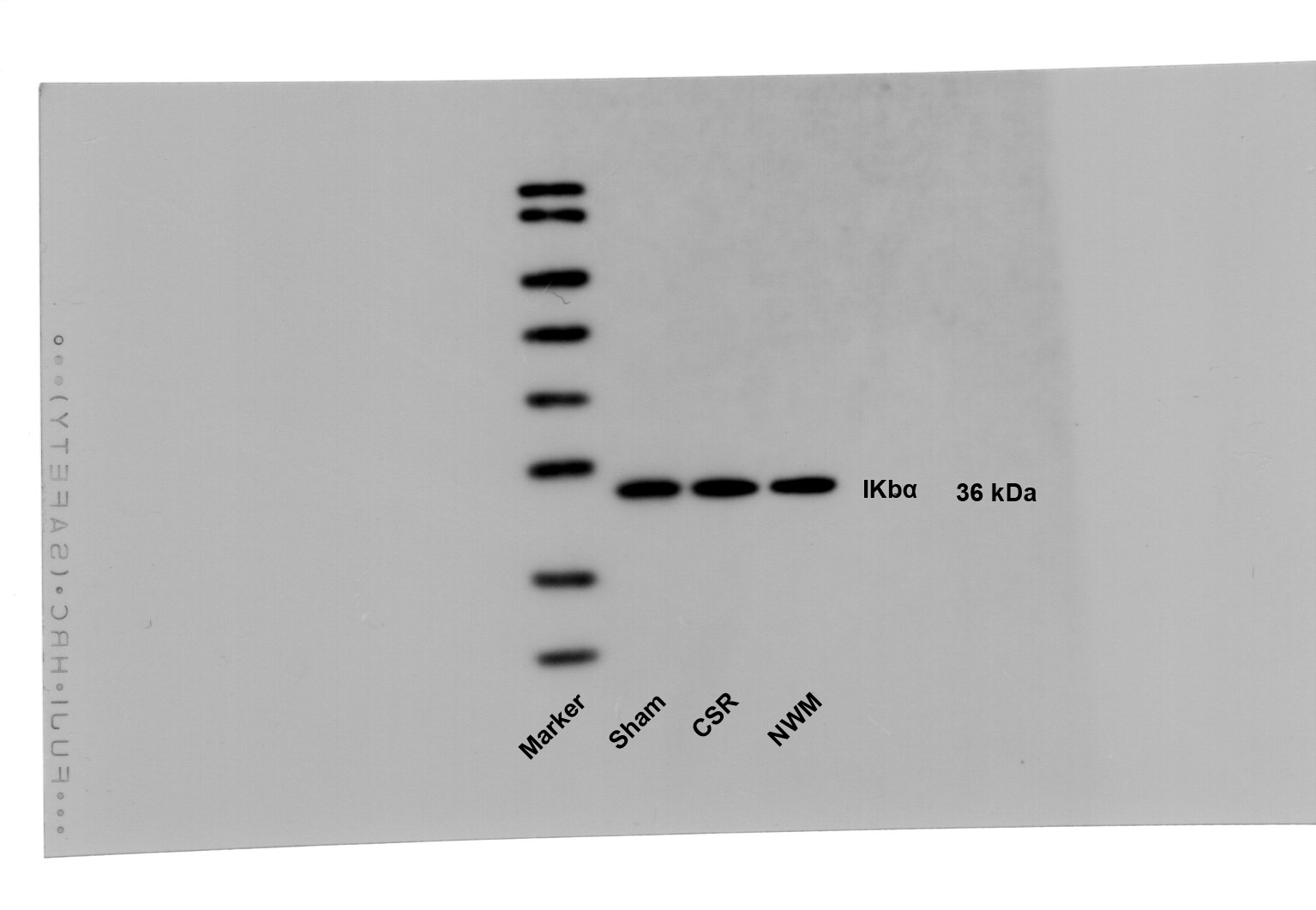

Supplement: Supplementary file 1 [file Data_Sheet_1.ZIP › 5A/11、IKBα.tif]

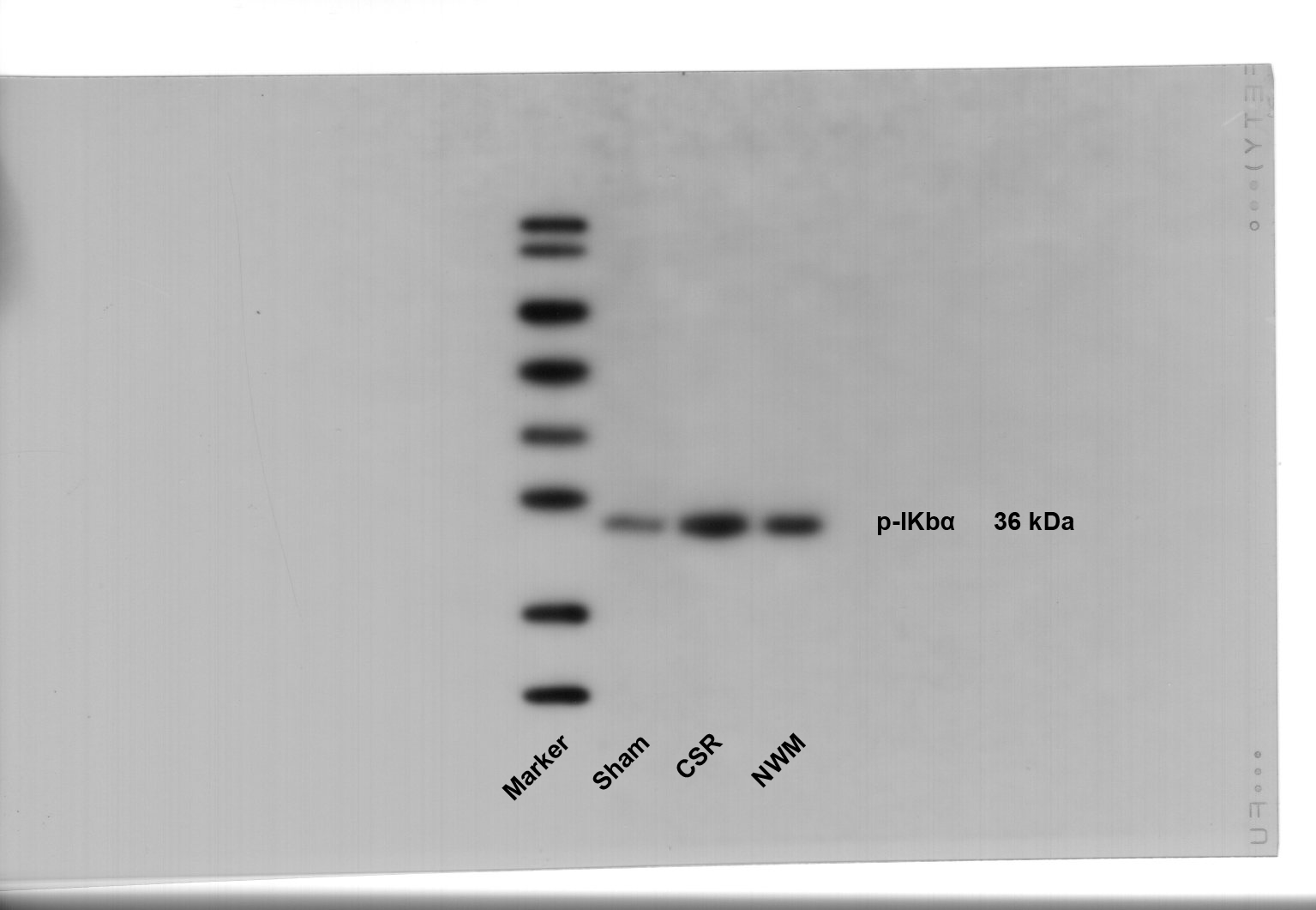

Supplement: Supplementary file 1 [file Data_Sheet_1.ZIP › 5A/12、P-IKBα.tif]

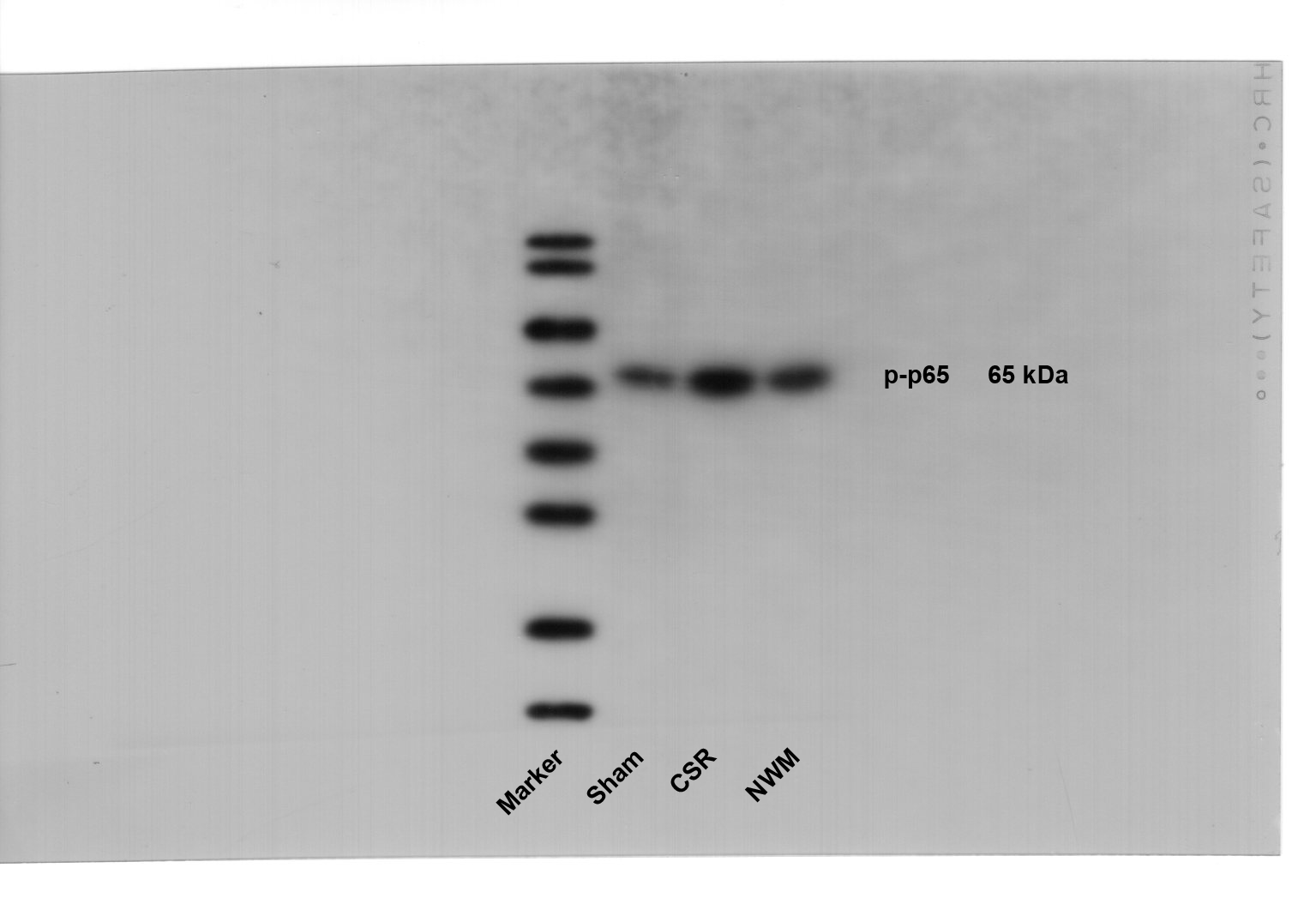

Supplement: Supplementary file 1 [file Data_Sheet_1.ZIP › 5A/13、P-P65.tif]

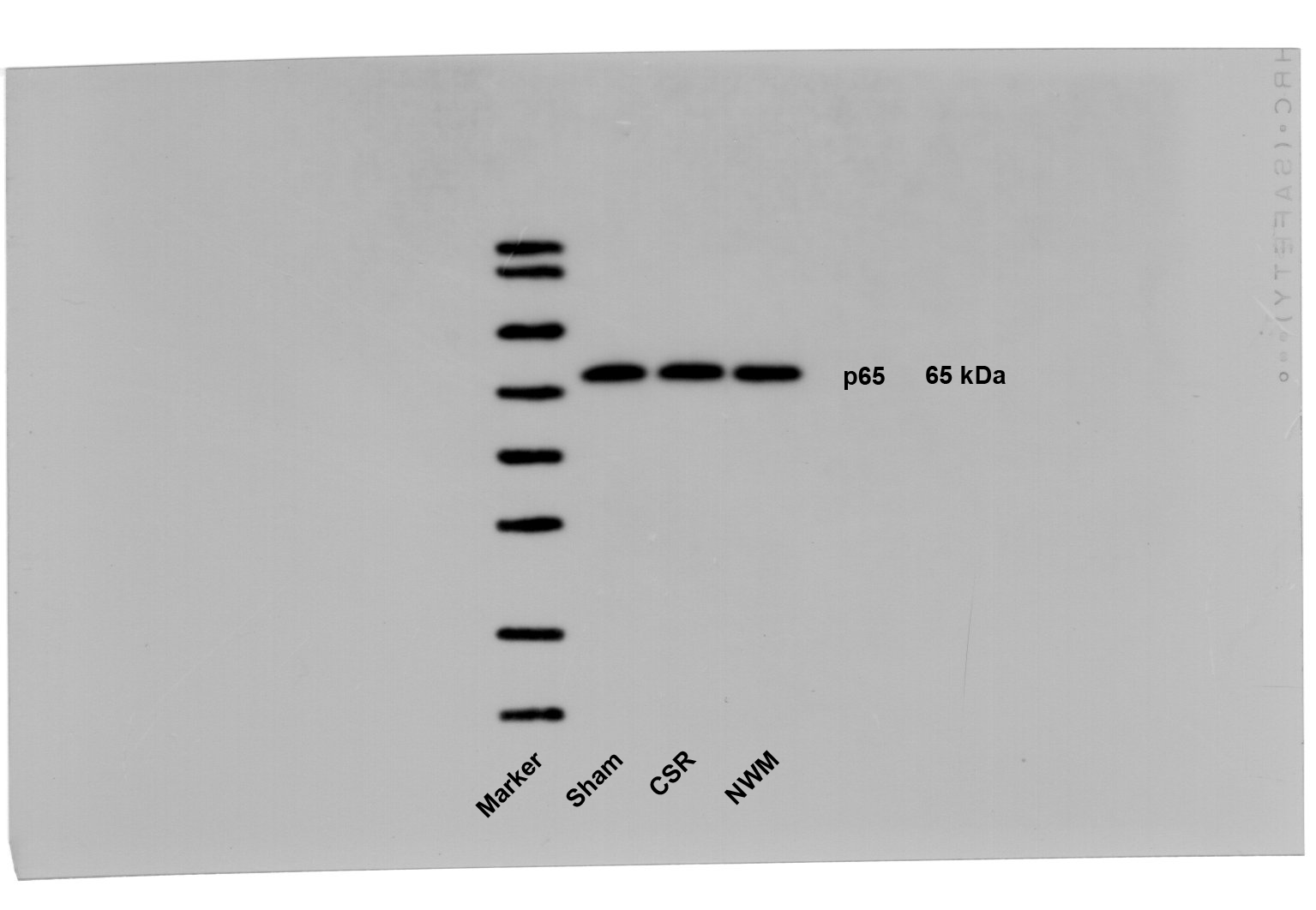

Supplement: Supplementary file 1 [file Data_Sheet_1.ZIP › 5A/14、P65.tif]

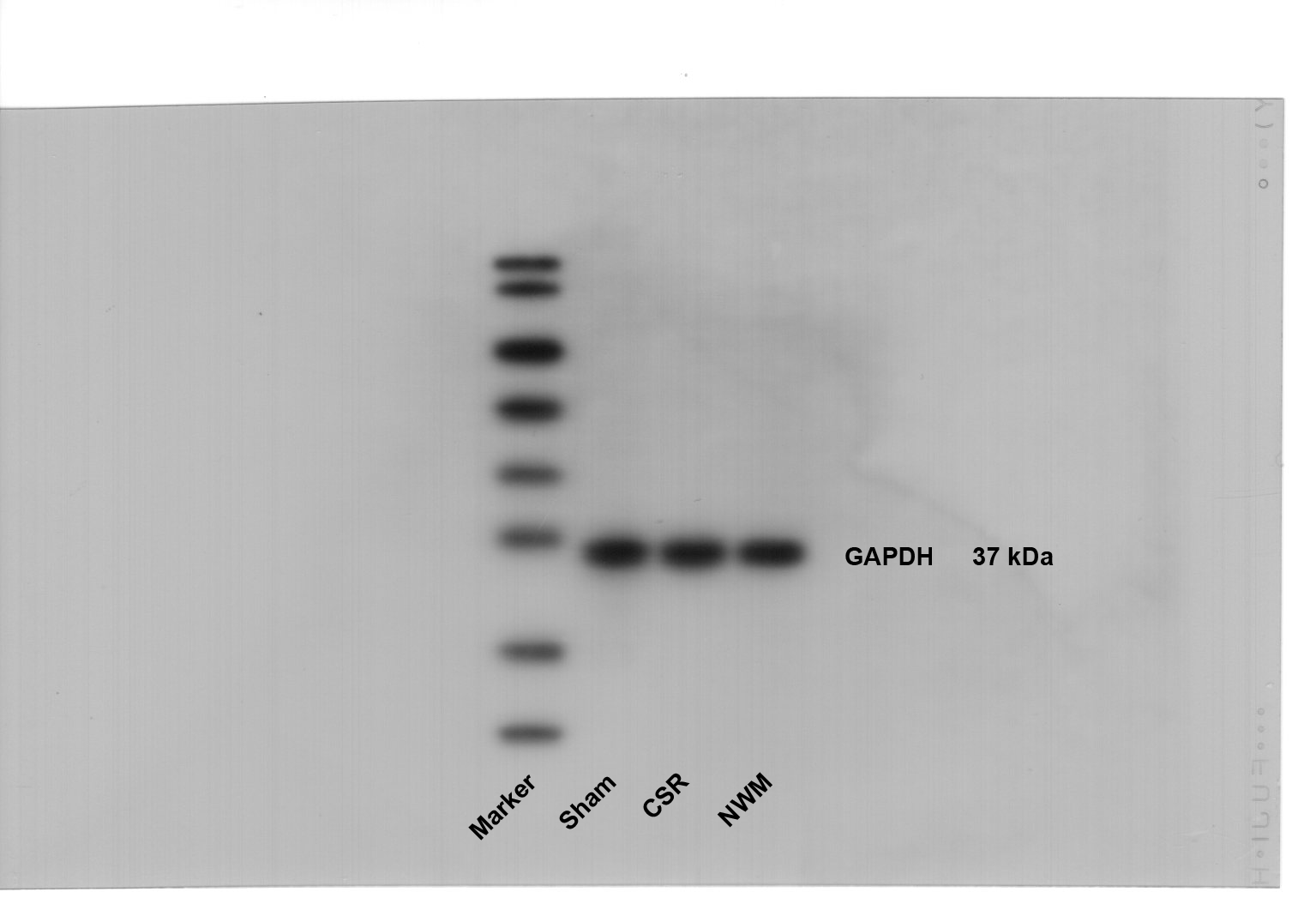

Supplement: Supplementary file 1 [file Data_Sheet_1.ZIP › 5A/15、GAPDH.tif]

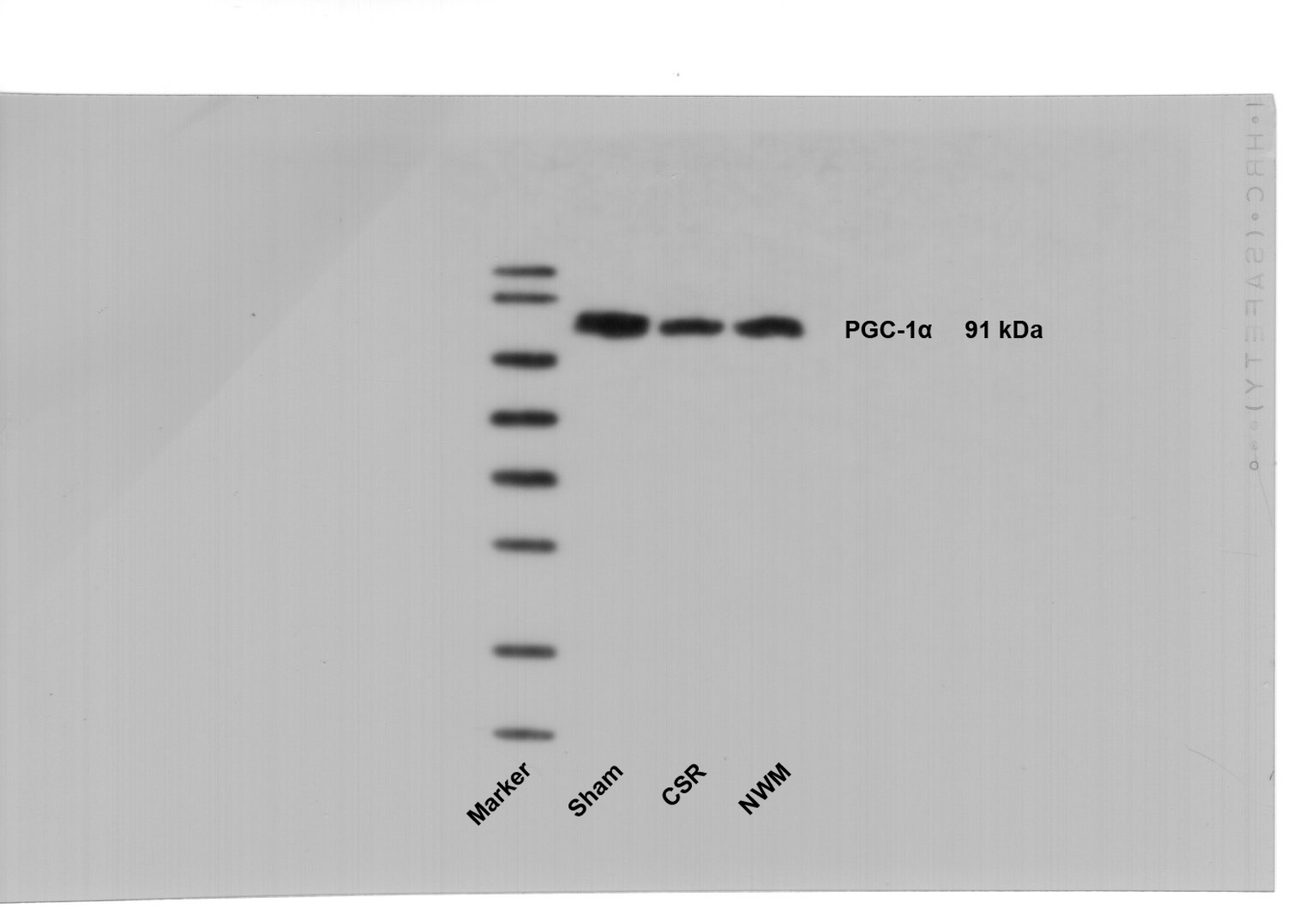

Supplement: Supplementary file 1 [file Data_Sheet_1.ZIP › 6G/16、PGC-1α.tif]

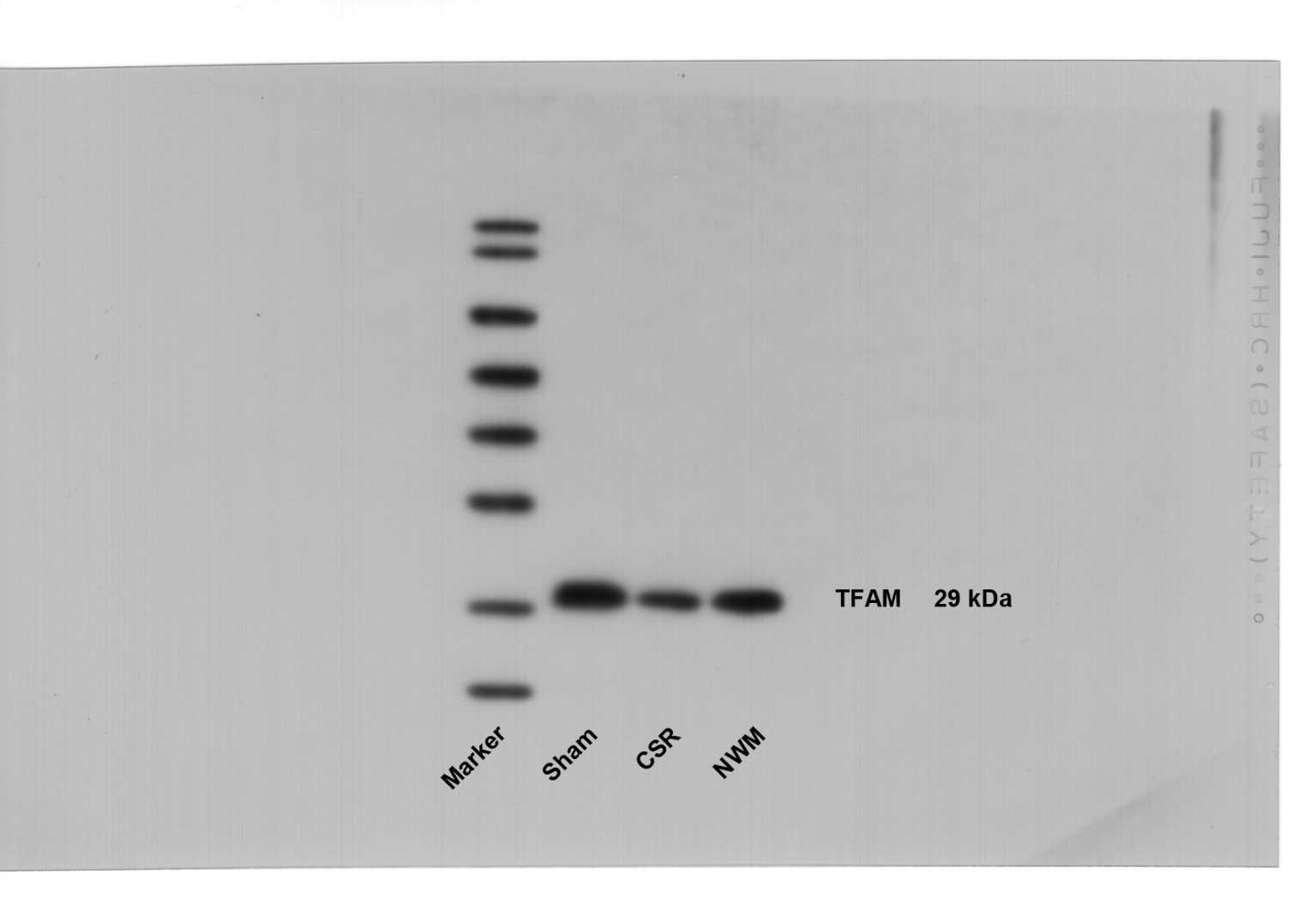

Supplement: Supplementary file 1 [file Data_Sheet_1.ZIP › 6G/17、TFAM.tif]

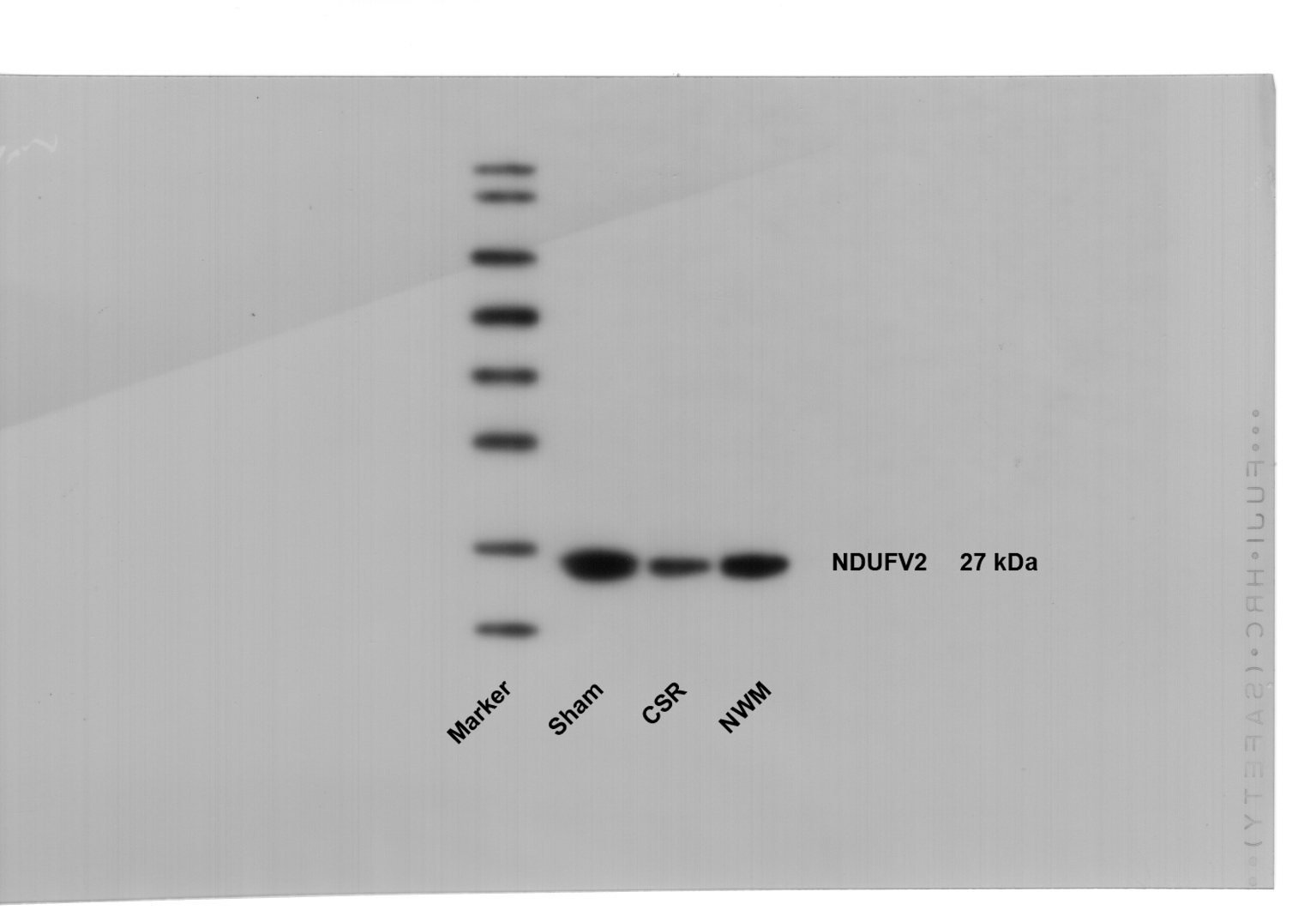

Supplement: Supplementary file 1 [file Data_Sheet_1.ZIP › 6G/18、NDUFV2.tif]

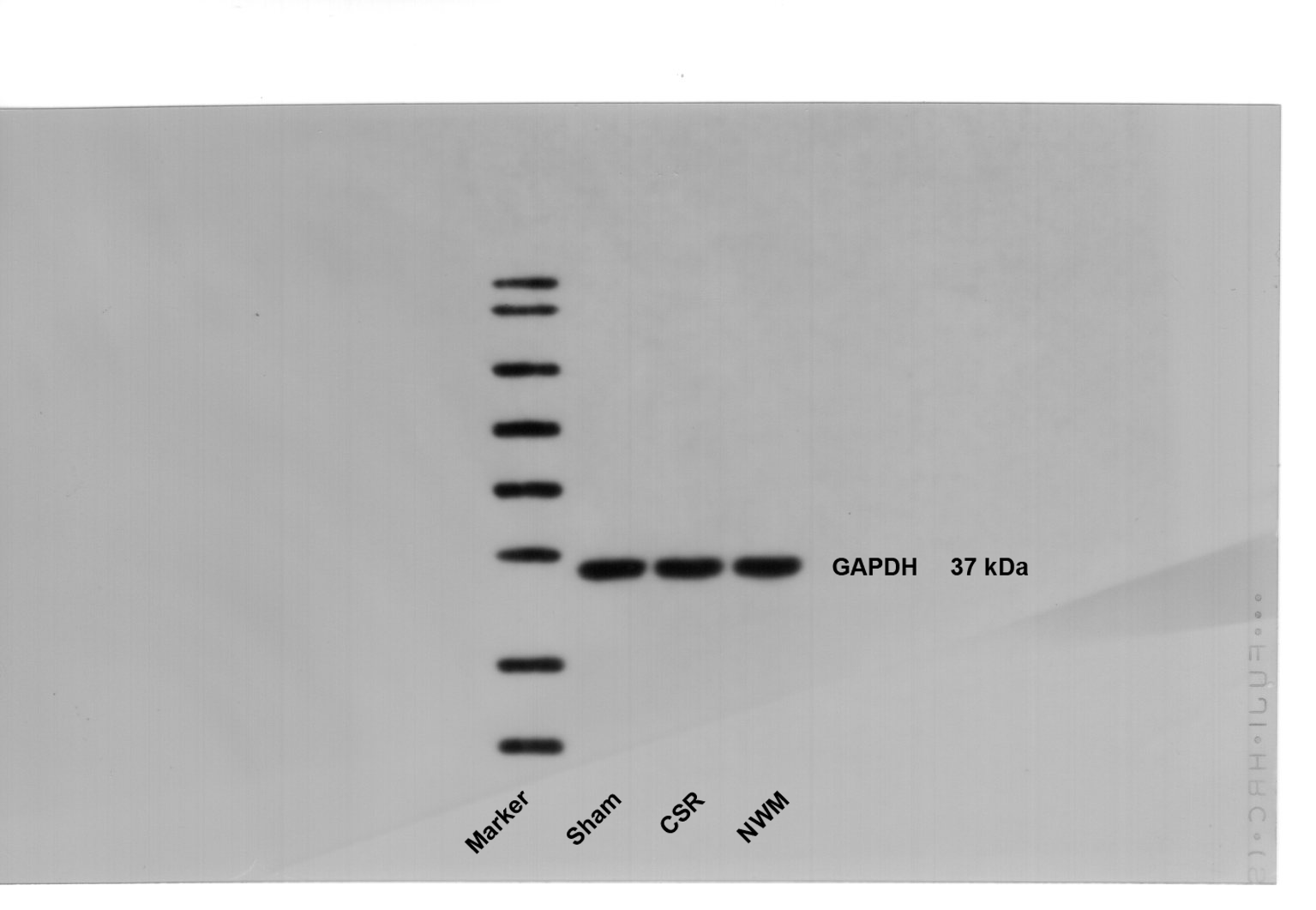

Supplement: Supplementary file 1 [file Data_Sheet_1.ZIP › 6G/19、GAPDH.tif]
